# Unlocking the Functional Properties of Plant Proteins in Designing Food Formulations for Senior Adults

**DOI:** 10.1111/1541-4337.70461

**Published:** 2026-04-21

**Authors:** Kinza Mukhtar, Jian Ying, Yong Wang, Cordelia Selomulya

**Affiliations:** ^1^ School of Chemical Engineering UNSW Sydney Sydney New South Wales Australia; ^2^ Nutrition and Health Research Institute, School of Life Science and Technology Wuhan Polytechnic University Wuhan China

**Keywords:** malnutrition, plant proteins, protein functionality, protein modification, senior adults

## Abstract

The aging population presents an increasing need for protein‐rich food that supports health, functionality, and quality of life in senior adults. Plant proteins, with their sustainability and nutritional potentials, are emerging as promising yet complex alternatives to animal proteins in this context. This review builds on recent advances in the functional properties, modification strategies, and formulation approaches of plant proteins tailored for senior adults. The discussion connects molecular and structural insights with nutritional relevance, showing how physical, chemical, and enzymatic modifications affect digestibility, texture, and bioactivity. However, these strategies can involve trade‐offs, whereby improvements in one functional property may compromise other functional attributes (e.g., aggregation decreasing solubility or extensive hydrolysis weakening gelation), highlighting the need to identify optimal processing windows. Aging‐related constraints, including dysphagia and anabolic resistance, are therefore discussed using measurable targets such as IDDSI‐aligned rheology/texture (e.g., viscosity, hardness) and nutritional metrics relevant to postprandial amino acid availability (e.g., leucine density and digestibility/bioaccessibility). By critically examining the interrelationship between processing, protein functionality, and physiological needs, this review provides an integrative framework for developing plant‐based foods to meet the specific dietary needs of senior adults.

## 1 Introduction

The global population of senior adults is increasing rapidly at an unprecedented rate, creating an urgent demand for foods that promote healthy aging and maintain physical and cognitive independence. Aging is a complex biological process marked by progressive declines in physical, physiological, and cognitive functions (Shaulson et al. 2024), where optimal nutrition plays a crucial role in delaying age‐related diseases and enhancing the quality of life for senior adults (Lee et al. 2021). According to the World Health Organisation, the number of people aged 60 years or older was 1 billion in 2020 and is projected to reach 1.4 billion by 2030 (Ageing and health, 2025). This trend is particularly pronounced in developed regions; middle‐income countries are also experiencing a significant increase in population size (Nakatani 2019). Poor dietary habits and nutritional deficiencies associated with aging contribute to chronic diseases such as type 2 diabetes, cardiovascular diseases, and malnutrition (Kaur et al. 2019). Reduced food intakes and impaired nutrient utilization further lead to the loss of muscle mass and bone density, which may increase the risk of sarcopenia, dysphagia, and osteoporosis, consequently (Sabir et al. 2023; Tangestani et al. 2020). Malnutrition is also correlated with a greater incidence of dementia and higher mortality and may exacerbate neurodegenerative processes through gut–microbiota–brain axis dysfunction (Bianchi et al. 2021). Collectively, these challenges highlight the multifactorial nature of malnutrition in senior adults, influenced by physiological decline, social circumstances, and lifestyle factors (Bardon et al. 2021).

Among the various forms of malnutrition, inadequate protein intake is particularly critical for senior adults (Kim et al. 2025). Protein synthesis is strongly influenced by the availability of essential amino acids, which must be supplied through dietary sources because they cannot be synthesized by the body. Adequate protein nutrition supports cellular functions and is recognized as a crucial lifestyle factor in preventing and managing muscle‐related issues such as sarcopenia (Cruz‐Jentoft et al. 2020). Greater protein intake is connected with improved muscle mass and strength (Cramer et al. 2016; Phillips and Martinson 2019), while it also contributes to bone health, influencing calcium balance and bone density (Groenendijk et al. 2023). Therefore, maintaining body protein levels is a primary nutritional goal for senior adults. The balance between net protein synthesis and degradation largely depends upon both the quantity and the quality of ingested protein (Kim et al. 2016). A well‐distributed and balanced protein intake can enhance muscle anabolism and help reduce muscle loss in senior adults (Moore et al. 2015). Consequently, higher and balanced protein intake is recommended for senior individuals over 65 with approximately 1.2 g/kg body weight per day (Siddique et al. 2017; Nowson and O'Connell 2015), and practical dietary strategies such as enriching regular foods and beverages have proven effective in achieving these targets, increasing daily intake by about 11.8 g/day to 1.14 g/kg/day (Beelen et al. 2017). Total protein intake refers to the absolute amount of protein consumed. Plant protein intake and animal protein intake refer to the amounts contributed by plant‐derived and animal‐derived foods, respectively, and together sum to total protein intake, which may also include other protein sources such as microorganism. Importantly, comparisons between plant and animal protein intake are often interpreted in a substitution context (i.e., increasing one source at a given total intake). Beyond the total amount of protein consumed, the nutritional value of protein is also determined by its quality (Adhikari et al. 2022). Protein quality varies among foods due to differences in amino acid composition, digestibility, the presence of antinutritional compounds, and the impact of processing methods (Addo et al. 2020; Han et al. 2015). These considerations are especially relevant for plant‐based diets, as plant proteins often exhibit lower digestibility and imbalanced indispensable amino acid profiles compared with animal‐derived proteins (Zhubi‐Bakija et al. 2021).

Meeting the growing protein demands of a rapidly expanding global population places increasing pressure on environmental and agricultural resources (Rulli et al. 2024). The high demand for animal‐based proteins, which are associated with substantial greenhouse gas emissions and intensive water and land usage, underscores the need for sustainable alternatives (Gerten et al. 2020), enhances the need for alternative protein sources. Alternative proteins are proposed to be a sustainable option for food security (Malila et al. 2024). Moreover, plant protein is recommended as healthier diet choice by the recent EAT‐Lancet commission (Rockström et al. 2025). Plant proteins, although widely considered as more sustainable than animal protein, often have lower nutritional quality due to their unbalanced amino acid profiles and low Protein Digestibility‐Corrected Amino Acid Score (PDCASS value; Bhandari et al. 2020). To enhance the plant protein quality, Dimina et al. (2022) used linear optimization of 151 plant proteins to create blends mimicking animal proteins, achieving up to 98.8% similarity to cow milk and 94.2% to egg white. Nevertheless, plant proteins usually face challenges in functionality, such as solubility and digestibility, which can hinder their effectiveness in food formulations. Therefore, modifying their functional properties is essential to improve digestibility as well bioaccessibility, particularly for senior adults. These modifications not only enhance the nutritional benefits but also improve plant proteins as valuable functional ingredients in food products by improving their rheological behavior and interfacial activity (Wang et al. 2025).

This review aims to provide a comprehensive understanding of the protein malnutrition challenges commonly faced by the senior adults and examines how targeted modifications of plant proteins might help to address these issues. Age‐related declines in digestion efficiency, swallowing ability, and muscle mass require food formulations that are specifically designed to the physiological needs of aging population. Accordingly, this review will explore the latest techniques for modifying plant proteins to enhance their functional properties, including digestibility, texture, and solubility, to improve suitability in elderly focused food formulations. By critically analyzing these developments, the review identifies key strategies for designing plant‐based foods that better support healthy aging and offers insights into future directions for advancing sustainable, nutrition‐oriented food innovation.

## 2 Nutritional Needs and Challenges in the Senior Adults

The population of senior adults face significant nutritional challenges, with malnutrition being one of the most prevalent and concerning issues. Leij‐Halfwerk et al. (2019a) analyzed 22 validated malnutrition screening tools for adults aged 65 and older. The findings showed that the prevalence of high malnutrition risk varied, ranging from 8.5% in community settings to 28.0% in hospitals across different countries. Malnutrition is not an inevitable outcome of aging, with various factors, such as geography, age distribution, and living conditions, contributing to this widespread problem. The prevalence of malnutrition can vary between 20% and 50% but increases in the population of senior adults from 29% to 61%. It has been demonstrated that the trend is higher in people over 60 years old (Kiesswetter et al. 2020).

**FIGURE 1:**
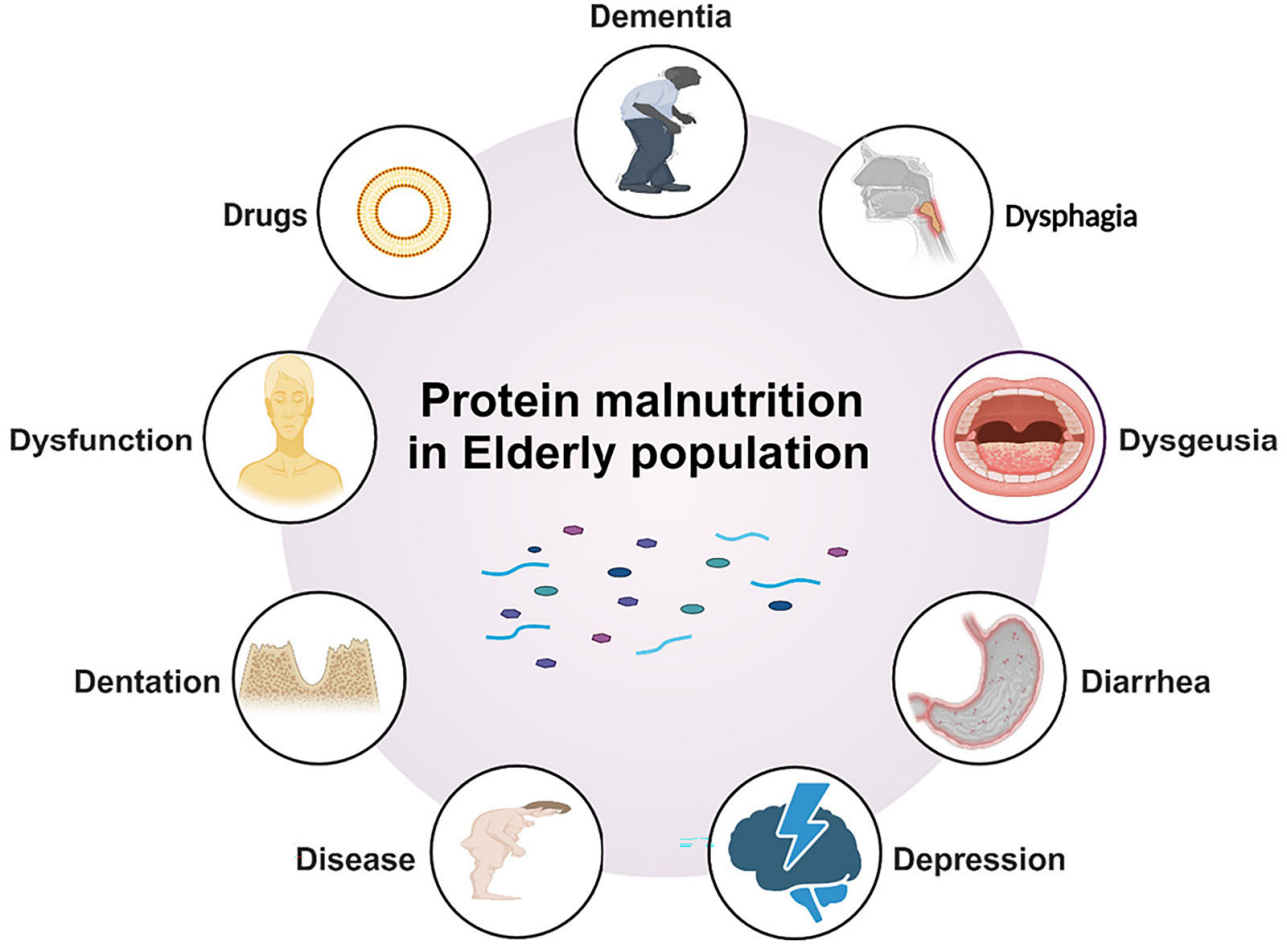
Key factors affecting senior health and nutrition (created in BioRender. https://BioRender.com/j35j927).

The term “protein malnutrition” was introduced in 1952 in Joint FAO/WHO Expert Committee on Nutrition (Protein Malnutrition, Proceedings of a Conference in Jamaica 1953). The causes of protein malnutrition in the senior adults are complex and involve a range of physiological, social, and economic factors, commonly known as the “nine D's” (dementia, dysgeusia, dysphagia, diarrhea, depression, disease, poor dentition, dysfunction, and drugs; Edington et al. 2000). Figure 1 illustrates the multifactorial causes of protein malnutrition in the senior adults, commonly referred to as the “nine D's.” Each of these elements contributes to reduced nutrient intake, impacting the overall health and nutritional status of senior adults (Ferrari et al. 2018). These physiological factors significantly impact food intake in senior adults. Senior adults in hospitals often do not meet their required protein intake (Ruiz‐Rosso et al. 2024). The protein consumption among senior adults at risk of malnutrition during hospitalization was only 0.65 g/kg/day, which refers to the median measured intake (Weijzen et al. 2020), and the below recommended thresholds of 1.0–1.2 g/kg/day for senior adults as outlined by clinical nutrition guidelines such as ESPEN and PROT‐age study group (Bauer et al. 2013; Deutz et al. 2014). Protein quantity, protein quality, and health outcomes represent related but distinct concepts. While adequate protein intake is important for meeting nutritional requirements in senior adults, evidence linking increased protein intake alone to improvements in muscle strength, physical function, or clinical outcomes remains inconsistent (Kirk et al. 2020). Functional outcomes are influenced by multiple interacting factors, including protein quality, digestibility, meal distribution, physical activity, and overall dietary context. In this review, statements regarding protein intake are therefore framed to distinguish intake adequacy from functional or health outcomes.

Malnutrition and serious illness often coexist, each exacerbating the other and affecting every organ system. The serious outcomes are mainly linked to the duration and extent of nutritional compromise. For instance, Rice and Normand (2012) stated that malnutrition can affect nutrient absorption, increase the complexity, and delay recovery. Dysphagia is a common issue in senior adults and can potentially decrease food intake and increase the risk of malnutrition. A meta‐analysis revealed that texture modification increased dietary energy and protein intake among adults with dysphagia (Chu and Chao 2025). In another study, high‐protein texture‐modified formulas were developed for senior people with dysphagia (23%–34% energy from protein) and observed improved swallowing capacity with these formulas versus standard commercial ones (Mongkolsucharitkul et al. 2025). Nutritional support for senior adults requires translation of age‐related clinical constraints into food structure and formulation parameters. Dysgeusia necessitates controlled flavor delivery and masking strategies, often achieved through emulsification and encapsulation systems, while impaired dentition highlights the need for reduced particle size and weakly structured protein gels to minimize mastication demands (Dellafiore et al. 2021). Dysphagia imposes rheological and textural requirements defined by the International Dysphagia Diet Standardisation Initiative (IDDSI), including controlled viscosity, yield stress, and cohesiveness, which can be engineered through plant protein thickening, gelation, and lubrication (tribological) functionalities (Wang et al. 2025). In addition, disease states may alter gastrointestinal conditions and digestion kinetics, underscoring the importance of designing protein structures that enhance protein bioaccessibility.

The consequences of malnutrition are broad, including conditions like sarcopenia and frailty, which are often interconnected (Volkert et al. 2019). Sarcopenia is considered part of frailty, but frailty is not necessarily a constituent of sarcopenia (Morley et al. 2013). Sarcopenia is progressive muscle loss, often linked to disease, inactivity, and inadequate protein intake, while frailty is reduced strength and physiological reserve that increases vulnerability and mortality (Seino et al. 2024). Protein intake and cognitive function are linked together, and studies concluded that high protein diets might play a role in better cognitive function in senior adults (Coelho‐Júnior et al. 2021; Glenn et al. 2019; Y. Li et al. 2020b).

## 3 Plant Proteins for Senior Nutrition

### 3.1 Role of Plant Proteins in Meeting Seniors’ Nutritional Needs

Plant proteins offer a viable alternative to animal proteins for meeting the nutritional needs of seniors, despite some limitations. Higher animal protein intake can mitigate malnutrition but also poses chronic disease risks (Berrazaga et al. 2019) because of saturated fatty acids (Health et al. 1992). In contrast, plant proteins are associated with lower levels of inflammatory biomarkers (di Giosia et al. 2022) such as C‐reactive protein, IL‐6, and fibrinogen (Pearson et al. 2003). Plant‐based diets are increasingly popular globally due to these and other potential health benefits (Craig et al. 2021), which depend on substitution pattern and overall diet quality. In 2022, the world vegan food market was valued at $16.53 billion, expected to increase with an 8.8% annual growth rate from 2023 to 2030 (Coherent Market insights 2024). Although plant‐based proteins can provide essential amino acids (Sá et al. 2020), they are often considered incomplete (Hughes et al. 2011). Although plant proteins play a vital role in meeting nutritional needs, an extensive knowledge of their functionalities is necessary to increase their applications in the food industry to replace animal‐based products (Sá et al. 2020).

The quality of dietary proteins varies, with plant proteins facing challenges related to amino acid composition, digestibility, the influence of the food matrix, and processing methods (Dardevet et al. 2021). Plant proteins typically have lower essential amino acid content, particularly in leucine, lysine, and sulfur‐containing amino acids such as methionine, and lower digestibility (Dardevet et al. 2021). Antinutrients, such as tannins, lectins, and phytates, also interfere with absorption, digestion, and protein utilization (Langyan et al. 2022). Despite these challenges, methodologies for calculating biological protein values often favor animal‐based proteins, particularly when total protein intake exceeds recommended levels (Craddock et al. 2021). Median protein intake is 1.23 g/kg/day, with essential amino acid requirements set at 0.214 g/kg/day by the Institute of Medicine, making up 17% of total protein intake (Craddock et al. 2021; Trumbo et al. 2002). Although some plant proteins have a lower essential amino acid content, such as cereals, however many pseudocereals and legumes contain higher values, and when combined, these plant sources can provide sufficient levels of essential amino acids to meet the necessary threshold for high‐quality protein intake (Craddock et al. 2021). In terms of protein quality, animal proteins, including milk, whey, casein, eggs, and beef, generally achieve PDCAAS values close to 1.0, indicating they provide all indispensable amino acids required for human growth and maintenance (Soh et al. 2024). These foods are therefore widely regarded as sources or complete protein. In contrast, plant proteins can be limited in one or more essential amino acids: legumes often contain lower amounts of sulfur‐containing amino acids such as methionine and cysteine, whereas lysine is typically the limiting amino acid in cereal grains (Young and Pellett 1994). Nevertheless, the content of limiting amino acids varies substantially among plant proteins. PDCAAS scores indicate that soy protein matches milk protein and whey with a PDCAAS of nearly 1.0, while canola, potato, pea, and quinoa proteins exhibit PDCAAS values of 0.75 or higher (Hertzler et al. 2020).

Research indicates that plant proteins can be as effective as animal proteins in addressing malnutrition with appropriate adjustments. Various analyses demonstrate that protein requirements are similar across individuals consuming animal, plant, or mixed diets. Meta‐analyses indicated no significant difference in protein requirements among these all‐people groups (Craddock et al. 2021). A study found that a 70%/30% plant‐to‐whey protein blend could activate muscle anabolism as effectively as whey protein alone if protein intake is increased by 25% per meal (Dardevet et al. 2021). Interaction analysis results suggest higher total protein intake is needed to see plant proteins’ beneficial effects on malnutrition. Nitrogen studies in humans revealed minimal differences in digestibility between animal and plant protein sources, with pea, wheat, and lupin flours having 89%–92% digestibility compared to eggs, meat, and milk proteins at 90%–95% (Craddock et al. 2021). Although both protein types benefit malnutrition, only plant protein is associated with improvements in inflammation‐related conditions, and antinutrients may positively impact health issues such as cardiovascular disease, cancer, and microbial infections (Langyan et al. 2022) such as saponins, have potential in lowering cholesterol and anti‐inflammatory properties (Ofori et al. 2022; Thakur et al. 2019). Even if animal proteins are considered higher quality, combinations of plant proteins or plant and animal protein blends can achieve similar biological values to animal proteins (Berrazaga et al. 2019; St‐Jules et al. 2019).

Food‐first dietary approaches, food fortification, and protein supplementation represent distinct nutritional strategies. Food‐first approaches prioritize meeting nutritional needs through habitual foods, while fortification enhances commonly consumed foods, and supplementation involves isolated nutrient products consumed in addition to the habitual diet. In senior adults, food‐first strategies are generally recommended, with fortification or supplementation considered when dietary intake is insufficient or impractical (Yu et al. 2024). In this manuscript, plant‐based protein ingredients are therefore discussed primarily within a food‐first and formulation‐based framework, rather than as direct substitutes for protein supplements.

Ensuring adequate nutrient intake through plant‐based fortification and supplementation is crucial for improving the quality of life and preventing chronic diseases among the senior adults. Existing literature shows that fortified food consumption and nutrient supplements can significantly improve nutrient intake and micronutrient status in senior adults (Kehoe et al. 2019). Food fortification relies on the “food‐first” principle, which means using energy‐dense and protein‐rich foods to enhance calories and protein intake, such as fortified bread, soups, and sauces (Tsikritzi et al. 2015; van Til et al. 2015). Studies suggest protein‐based supplementation and fortification are effective interventions to enhance dietary intake in senior adults (Mills et al. 2018; Morilla‐Herrera et al. 2016). A 10‐week study with 46 senior participants with early sarcopenia showed a significant increase in appendicular skeletal muscle mass index and a reduction in low‐density lipoprotein levels (Chang et al. 2023). A national survey reported that 70% of senior adults consume fortified products, significantly contributing to their intake of vitamins D (18%), folate (18%), B6 (17%), and E (15%), with smaller contributions to vitamins C, B1, B2, calcium, and iron (4%–6%; Ocké et al. 2013). While these findings support the broader role of food fortification in addressing nutritional inadequacies, evidence specific to plant protein fortification and related functional or health outcomes remains limited. Therefore, plant protein fortification can also play a crucial role in meeting the nutritional requirements of senior adults.

### 3.2 Plant Protein Modifications to Improve Their Functionality in Food for Senior Adults

Addressing malnutrition in senior adults requires developing foods tailored to their specific nutritional needs, recognizing that health outcomes depend not only on nutrient content but also on the functionality of food components (Aguilera 2006). Establishing a proper food structure influences the nutritional efficacy of nutrients during digestion (Somaratne et al. 2020). Therefore, designing food for the senior adults should integrate structural and formulation approaches, ensuring that products are both palatable and beneficial, while maintaining quality standards (Alongi and Anese 2021).

Plant‐based proteins can support healthy aging by reducing risks linked to animal protein intake (Gueugneau 2023). Plant proteins face challenges such as low solubility, limited digestibility, and reduced bioavailability, which restrict their broader application. Enhancing functional properties like solubility, surface hydrophobicity, and digestibility offers a promising route to improve their nutritional effectiveness and potential contribution to healthy aging in senior adults. Modifying plant proteins through different treatments enhances their functional properties, allowing for their application in food products designed to meet dietary needs of senior adults, such as bioavailability and digestion. Plant proteins’ functional properties, such as solubility, have important critical in determining their functionality and bioavailability. For instance, large aggregates in the gastrointestinal tract can limit the digestive enzymes’ access to the protein, hence reducing protein digestibility and bioavailability (Amigo and Hernández‐Ledesma 2020). Emulsification, foaming, and gelation help to enhance the sensory and nutritional qualities of different food formulations. For instance, the excellent foaming capability is vital for achieving a fluffy texture in items like cakes and ice cream, with plant albumin molecules forming stable interfacial layers around air bubbles (Alongi and Anese 2021), which could be beneficial for the safe swallowing of senior adults. Gelation is another property of plant proteins, crucial for creating elastic foods like custards/pudding and yogurts, which can be induced through various methods, including heat, pressure, chemical, and enzyme treatment (Jack Yang et al. 2022a). For instance, soybean protein is particularly notable for its gelation properties due to globulin composition, heat stability, as seen in tofu production, which involves the coagulation of soy milk (Zhao et al. 2016). Factors such as plant protein variety and processing methods significantly influence gel quality (Chen et al. 2017). In baking, plant protein, water‐holding and retention properties play a crucial role in bread structure and storage (Hoehnel et al. 2019).

To enhance the suitability of plant proteins for diverse food applications, modifications are applied to improve their functional properties, which include their roles and characteristics in various processes and interactions with other components (D'Alessio et al. 2022; Mir et al. 2020). These characteristics include solubility, emulsification properties, water‐holding capacity, allergenicity, gelation, and foaming ability which are functions of protein structure and composition. For example, proteins with high hydrophilic regions exhibit high solubility (Schein 1990), while emulsification properties rely on the amphiphilic nature of proteins (Hoffmann and Reger 2014). Similarly, gelation depends on conformational alterations in secondary and tertiary structures (El‐Anany et al. 2023). Protein functionality is influenced by various factors, including intrinsic ones such as amino acid composition and structure and extrinsic elements like temperature and pH (Singh et al. 2023). Understanding these aspects is crucial for improving protein functionality and food applications.

**FIGURE 2:**
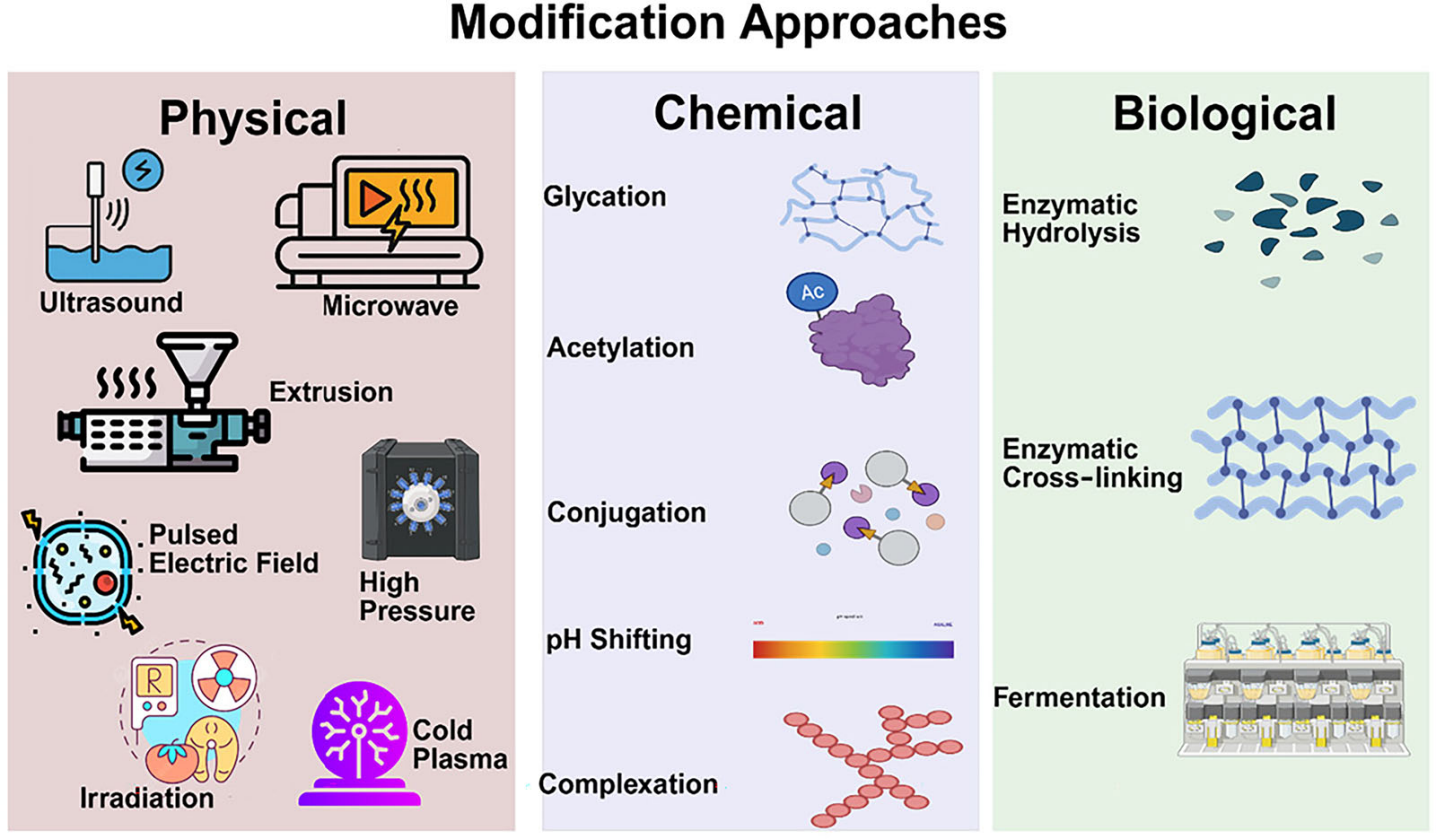
Protein modification approaches: A breakdown of physical, chemical, and biological methods (created in BioRender. https://BioRender.com/b99l202).

Due to the inherent challenges in utilizing plant proteins effectively, it is crucial to explore and implement physical, chemical, and biological modifications. These methods allow for targeted alterations in protein structure, thereby enhancing key functional properties such as solubility, digestibility, and emulsification. However, these modification strategies often require optimum conditions; improvements in one property may compromise other functional attributes, such as an increase in surface hydrophobicity (excessive unfolding), which could decrease solubility, while partial unfolding favors the system. By applying these modification techniques, it becomes possible to enhance plant proteins for broader applications in food products and bridge the gap between their current limitations. Figure 2 illustrates the various physical, chemical, and biological modification techniques that alter protein structures and functionalities. Physical methods include ultrasound, microwave, extrusion, irradiation, cold plasma, and high pressure, while chemical approaches involve glycation, acetylation, pH shifting, and complexation. Biological modifications, such as enzymatic hydrolysis and cross‐linking, along with fermentation, are also shown to influence protein properties for improved functionality. These modifications pave the way for innovative applications of plant proteins in improving nutritional solutions, discussed in the next section.

## 4 Physical Modifications

Physical modifications refer to mechanical or thermal treatments applied to alter the structure and functionality of proteins without using chemicals or biological processing. These methods are particularly valuable for enhancing the functional properties of plant‐based proteins, such as solubility, emulsification, digestibility, and texture. Table 1 outlines the treatment conditions used for different protein types and highlights the resulting changes in physical properties, functional properties, molecular structure, and digestibility.

**TABLE 1:** Impact of physical modifications on functionalities of plant proteins.

Modification approach	Treatment conditions	Protein type	Physical properties	Functional properties	Molecular structure	Digestibility and bioaccessibility	References
Conventional heating Ohmic heating	80–100°C, 30 min	Album (*Chenopodium album*)	—	↑ Surface hydrophobicity ↑ Thermal Stability ↑ Gelling ability	—	↑ In vitro digestibility (from 77% to 87.5%),	Mir et al. (2020)
	65, 75, 85, and 100°C 30 min	Pea and soy	—	↑ Surface hydrophobicity		↑ In vitro digestibility (from 30% to 60%)	Tang et al. (2022)
	100°C, 60 min	Rice	↑ Solubility (by two times) ↓ Particle size (from 325 m to 225 nm)	↑ Foaming ability (by three times)	—	—	Zhao et al. (2020)
	75–175°C, 60 min	Faba bean	—	↑ Water‐holding capacities (to 113%) ↑ Fat binding capacities (to 116%)	—	—	Bühler et al. (2020)
	75°C, 85°C, and 95°C, 5 min	Soy	↓ Solubility	↑ Surface hydrophobicity ↑ Viscosity ↑ Emulsifying ability ↓ Gel strength	—	—	Zhang et al. (2024)
	76.8–91°C, 20 min	Soy	↓ Particle size (from 181.97 to 167.47 nm)	—	—	—	Shen et al. (2022)
	5 V/cm	Lentil	—	↑ Surface Hydrophobicity	↑ β‐sheets	—	Miranda et al. (2023)
	50, 75, 100 and 125 V/cm for 4 min	Peanut	—	↑ Cohesiveness in texture	—	—	Y. Chen et al. (2023b)
	25 V cm^−1^ (90 ‐150°C)	Pea	↑ Solubility (by 240%)	—	↑ *β* sheet	—	Avelar et al. (2024)
Microwave High‐pressure processing	600 W, 1 min	Soy	—	↑ Water‐holding capacity (85.64% ± 0.93%) ↑ Gel strength (145.41 ± 6.18 g)	—	—	Mu et al. (2020)
	350 W, 120 min	Sunflower	↓ Particle size (from 17.40 to 16.03 µm) ↑ Solubility (from 72.14 ± 0.04% to 74.50 ± 0.01%)	↑ Emulsion stability (by 1.43 times)	—	—	Gultekin Subasi et al. (2022)
	560 W, 3min	Quinoa	↑ Solubility (from 17.7% to 81.5%)	↑ Emulsifying ability ↑ Gelling capacity	↓ α‐helix ↑ β‐sheet	—	L. Wang et al. (2021b)
	180 W, 4min	Pea	↑ Solubility (by two times)	—	—	—	Ertugrul et al. (2021)
	800 W, 2min	Jack bean	↑ Solubility (82%)	↑ Surface hydrophobicity ↑ Protein yield ↑ Gelling ability ↑ Emulsion activity (144.23 m^2^/g) ↑ Foaming ability (59.30%) ↑ Water‐holding capacity	—	—	Ajayi et al. (2024)
	30 and 50 MPa, 38–40°C	Pea	↓ Particle size (from 24.20 to 0.38 µm), ↑ Solubility (from 37% to 95%) ↑ Turbidity	↑ Surface hydrophobicity ↑ Foaming capacity (from 15% to 34%) ↑ Emulsifying capacity (from 61% to 74%)	—	—	Lijuan Luo et al. (2022b)
	70, 100, and 150 MPa, 30°C	Pea	↓ Particle size (47–45 nm) ↑ Solubility (from 22% to 34%),	↑ Water‐holding capacity (from 196% to 230%) ↑ Oil‐holding capacity (from 149% to 252%)	—	↑ Digestibility	Melchior et al. (2022)
	60 and 100 MPa, five cycles	Pea	↑ Particle size (92.08 ± 5.23 µm) ↓ Solubility	↑ Water‐holding capacity ↑ Oil‐holding capacity	↓ β‐sheet	—	D'Alessio et al. (2023)
	50 MPa, three cycles	Pea	↓ Particle size ↑ Solubility (by three times)	↑ Surface hydrophobicity ↑ Foaming ability ↓ Foaming stability ↑ Emulsifying activity ↓ Emulsion stability	↓ β‐sheet	—	Yan et al. (2024)
	30, 60, 90, 120, or 150 MPa, three cycles	Chickpea	↓ Particle size (by 72.6%) ↑ Solubility (to 94%) ↓ Turbidity	↑ Surface Hydrophobicity ↑ Foaming capacity ↑ Emulsion activity	—	—	Ma et al. (2023)
	30, 60, 90, 120, and 150 MPa, three cycles	Quinoa	↓ Particle size (by 94%) ↑ Solubility (to 52%) ↓ Turbidity (by 65%),	↑ Zeta potential (from 15.77 mV to (43.73 mV) ↑ Emulsifying activity (by 39%) ↑ Emulsifying stability (by 186%) ↑ Foaming capacity (by 100%) ↑ Foaming stability (by 185%)	—	—	Lan Luo et al. (2022a)
	0, 10, 30, 70, and 100 MPa, two cycles	Oat	↓ Particle size ↑ Solubility	↑ Surface hydrophobicity ↓ Zeta potential ↑ Emulsion stability	—	—	Li et al. (2024)
	0, 30, 60, 90, and 120 MPa, two cycles	Hemp	↓ Particle size ↑ Solubility (up to 48.7%)	↑ Surface hydrophobicity	↓ α‐helix ↓ β‐sheet ↓ β‐turn ↑ Random coils	—	Qingling Wang, Tang, et al. (2024c)
	130 MPa with three cycles	Hemp	↓ Particle size (from 365 to 182.8 nm) ↑ Solubility (1.44‐fold)	↑ Emulsion activity ↑ Emulsion stability	—	↑ In vitro digestibility (from 84.23% to 91.43%)	Karabulut et al. (2024)
	25, 50, 100, and 200 MPa, up to 10 cycles	Lupin	↓ Particle size (from 54 to 1.60 µm) ↑ Solubility (up to 95%)	—	—	—	Lo et al. (2024)
Ultrasound	100–400 W, 5 min	Pea	↑ Solubility (from 7.2 to 58.4 mg/mL)	↓ Foaming ability	↓ α‐helix ↑ β‐sheet	—	Gao, Rao et al. (2022a)
	400 W, 16 min, 25 kHz	Soy	—	—	↓ α‐helix ↑ β‐sheet and Random coil	↑ Digestibility (up to 93%)	Vanga, Wang and Raghavan (2020b)
	500 W, 20 kHz, 5 min	Soy, Pea, Oat, Rice	—	—		↑ Digestibility. ↑ Bioaccessibility by (5.59% for rice, 4.16% for oat, 8.78% for corn, and 18.69% for soy)	Yuanqing et al. (2020)
	200–600 W, 5 min	Soy	↑ Solubility (up to 80%)	–	↓ *α*‐helix and *β*‐turn ↑ β‐sheet ↑ Random coil contents		Yan et al. (2021b)
	500 W, 15 min, 25°C	Soy	↓ Particle size ↑ Solubility ↓ Turbidity	↓ Surface hydrophobicity ↑ Emulsifying activity ↑ Emulsion stability ↑ Foaming ability ↑ Foaming stability ↑ ζ‐potential	↑ α‐helix and β‐sheet	—	M. Wang et al. (2024a)
	20 kHz, 60%–100% amplitude, Power intensity 55–105 w/cm^2^ 5–30 min	Buckwheat	↑ Particle size (from 427.7 ± 76.7 nm to 2130.8 ± 356.2 nm)	—	↓ β‐turn and β‐sheet (by 40.9% and 22.4%) ↑ Random coil, and α‐helix (by 30.6%, and 25.5%)	↑ Digestibility (from 41.4% to 58.2%)	Jin et al. (2021)
	20 kHz, Power unspecified, 1–16 min	Almond	—	—	↑ Random coils (From 14.34% to 19.47%)	↑ Digestibility	Vanga, Wang, Orsat et al. (2020a)
	400 W, 20 kHz, 50% duty cycle, 0–16 min	Kiwifruit	↓ Solubility (by 20%)		↓ α‐helix ↑ β‐sheets	↑ Digestibility (up to 77%)	Wang et al. (2021a)
	37 W/cm^2^, 7.8 min	Hemp	↓ Particle (from 180.5 nm to 98.2 nm) ↑ Solubility (from 20.3% to 36.8%)	↑ Foaming ability (from 102.5% to 163.5%) ↑ Emulsifying ability (from 21.45 m^2^/g to 28.14 m^2^/g)	—	—	Karabulut and Yemiş (2022)
	200–600 W, 10–30 min	Potato	↓ Particle size (by 8.72%–54.03%) ↑ Solubility (from 0.778 ± 0.311 mg/mL to 12.66 ± 0.412 mg/mL)	↑ Emulsifying ability (27.83 m^2^/g)	↑ β‐sheet ↓ random coil and β‐turn	—	Zhao et al. (2022)
	500 W, 5–30 min	Lupin	↓ Particle size pH 5 (from 55 µm to 10 µm) pH 9 (from 15 µm to < 1 µm)	—	—	—	Lo et al. (2022)
	0–600 W, 30 min	Tiger nut	↓ Particle size (246.4–160.7 nm) ↑ Solubility (from 41.29% to 67.53%)	↑ Emulsifying ability	↓ α‐helix and β‐turns ↑ β‐sheet ↑ Random coil structures	—	Cui et al. (2021)
	100 W‐500 W, 20 kHz, 30 min	Pumpkin	↑ Solubility	↑ Emulsifying ability	—	—	Du et al. (2022)
	100, 200 W, 5–10 min	Cowpea	↑ Solubility (from 57.26% to 68.85%)	↑ Foaming ability (from 70.64% to 83.74%) ↑ Emulsifying ability (from 47.48% to 64.26%)	—	↑ Digestibility (from 88.27% to 89.99%)	Loushigam and Shanmugam (2023)
	Tri‐frequency (28/40/50 kHz)	Qingke (Tibetan hulless barley)	↑ Solubility (by (43.54%)	↑ Foaming ability (by 20.83%)	—	—	Chen et al. (2023a)
	200, 400, or 600 W, 15 and 30 min	Almond	↓ Particle size (from 20.01–4.97 µm) ↑ Solubility (from 60.4% to 95.2%)	↑ Emulsifying ability (from 12.8 to 37.4 m^2^/g) ↑ Foaming ability (from 56% to 94%)	↓ α‐helix and β‐turns ↑ in random coils	—	Tian et al. (2024)
	150 W, 5, 10, and 20 min	Pumpkin	↑ Particle dispersibility ↑ Solubility (by 20 points) ↓ Turbidity	↑ Emulsifying activity index (to 73%–82%) ↑ Foaming ability (from 30% to 55%)	—	—	Ramondo et al. (2024)
	Power 400 W, Amplitude (60%–90%), 30 min	Faba Bean	↑ Solubility (from 74.38% to 90.98%),	↑ Surface hydrophobicity (by 40.53%) ↑ Emulsifying activity (by 49.52%) ↑ Foaming ability (twofold) ↓ ζ‐potential,	↓ α‐helix content, ↑ β‐sheet content	—	Gulzar et al. (2024)
	250 W, 20 kHz, 1, 3, 5 and 10 min	Faba bean	↓ Particle size (from 370.47 to 187.87 nm) ↑ Solubility (89.11%)	↓ Zeta potential (from − 40.83 to − 37.17 mV)	—	—	Adal (2024)
	200 W, 400 W, and 600 W, 15 and 30 min	Soursop seed	↓ Particle size ↑ solubility (from 109% to 260%)	↑ Water‐holding capacity (to 38.4%) ↑ Emulsifying activity index (to 86%) ↑ Foaming capacity (to 41%) ↓ Gelation concentration	—	—	López‐Mártir et al. (2024)
Cold atmospheric plasma	80–100 Hz, (1–10 min)	Soy	↓ Particle size ↑ Solubility (from 13.2%–50%)	↑ Emulsifying ability (from 56% to 168%) ↑ Foaming properties (from 60% to 194%)	—	—	Zhang et al. (2021b)
	70.0 W (35 V, 2A) for 3.0 min	Pea	—	—	—	↑ Bioaccessibility	Yu et al. (2021)
	18 kV for 5, 10, and 15 min	Soy	↑ Solubility	↑ Gel strength ↑ Springiness ↑ Water‐holding capacity ↓ Emulsifying ability	↑ β‐sheet ↓ α‐helix, β‐turn and random coil,	–	Zeinali and Soltanizadeh (2024)
	500, 600, 700, and 800 W 4.5 min	Pea	↑ Solubility (by 25.11%)	↑ Emulsifying activity (20.22 m^2^/g) ↑ ζ‐potential ↑ Surface Hydrophobicity	↓ α‐Helix and β‐sheet ↑ β‐turn and Random coil	–	Ji et al. (2024)
	50, 60, and 70 kV for 60 s	Coconut	↓ Particle size	–	↓ α‐helix content and β‐sheet	↑ Digestibility	Chen et al. (2024)
	50 W, 0, 1, 2, 3, 4 and 5 min	Sunflower	↓ Particle size ↑ Solubility	↑ Zeta potential ↑ Surface Hydrophobicity ↑ Emulsifying activity (by 83.9%)	↓ α‐Helix ↑ β‐Sheet and β‐turn	–	P. Wang et al. (2024b)
	1 kV and 2 kV for 5, 15, and 25 min	Millet	↓ Particle size (from 605.70 to 247.00 nm) ↑ Solubility (60.61%–82.08%)	↑ Zeta potential ↑ Surface hydrophobicity ↑ Water‐holding capacity ↑ Emulsifying activity (from 42.94 to 75.98 m^2^/g),	–	–	Monica et al. (2024)
Pulsed electric field	25 kV/cm, Pulse numbers (0–400)	Mung bean	↑ Solubility	↑ Surface hydrophobicity ↑ Emulsifying ability	–	–	Gulzar et al. (2023)
	5, 10 and 20 kV/cm, Pulse numbers (6.42)	Soy	↓ Particle size ↑ Solubility	↑ Surface hydrophobicity ↑ Emulsifying activity (from 26.02 m2/g to 30.44 m^2^/g)	↓ α‐Helix and β‐sheet ↑beta‐turns and Random coil	–	Wang et al. (2023a)
	1.5 kV/cm, Pulse numbers (1000–4000)	Faba	↓ Particle size (to half) ↑ Solubility (82.12 ± 1.99%)	↑ Surface hydrophobicity (28.74%) ↑ Emulsifying activity index (by 36.13%) ↑ Foaming ability (by twofold) ↓ζ‐potential	–	–	Gulzar et al. (2024)
Extrusion	Dry feed rate (2.75 kg/h) and final extrusion feed moisture content (FMC) of (38% or 42%), Screw speed (350–450 rpm)	Pea	–	↑ Water‐holding capacity ↑ Oil absorption capacity ↑ Emulsion capacity (70%–82%) ↑ Emulsion stability	–	–	Chan et al. (2024)
	Feed rate 1.4 kg/min, 140–190°C, Screw speed 30 rpm	Soy	–	↑ Springiness and Chewiness	–	↑ In vitro digestibility (63.8 ± 0.5%)	Fu et al. (2024)
Irradiation	7.5 kGy	Soy	↑ Solubility (23 ± 0.21%)	↑ Foaming ability (62.5 ± 0.34%) ↑ Foaming stability (41 ± 0.47%)	–	–	Yuying Wang et al. (2020a)
	2 kGy	Rice	↑ Solubility (69.18 ± 1.07%), –	↑ Water‐holding capacity (5.89 ± 0.08 g/g) ↑ Oil‐holding capacity (3.45 ± 0.04 g/g) ↑ Emulsifying activity (45.65 ± 1.26 m^2^/g)	↑ β‐Sheets (31.16 ± 0.16) ↑ Random coil content (14.56)	–	Yao et al. (2022a)

(↑) indicates increase; (↓) decrease; (–) not available in the literature studies.

### 4.1 Thermal Treatment

Heat treatment is widely used to modify protein foods by breaking hydrophobic, electrostatic, hydrogen, and disulfide bonds, especially above denaturation temperatures (Aryee et al. 2018). Controlled heat may improve gelation (Sun and Arntfield 2011), emulsification (Peng et al. 2016), and digestibility (Rehman and Shah 2005) of plant proteins, but can reduce solubility due to aggregation (Sun‐Waterhouse et al. 2014). The effects depend on temperature, rate, ionic concentration, and pH (Zink et al. 2016).

Heat treatment significantly alters the structural and functional properties of plant proteins, enhancing their suitability for various food applications. Across different protein sources, conventional heating (75–175°C) has been shown to improve functional attributes such as surface hydrophobicity, emulsifying ability, and thermal stability, while also affecting solubility and particle size. For instance, heat‐treated soy and pea proteins exhibited reduced particle size and enhanced emulsifying properties (Chao and Aluko 2018; Shen et al. 2022; Q. Li et al. 2020a). While faba bean and rice proteins demonstrated increased water and fat binding capacities, solubility, and foaming ability (Bühler et al. 2020; Zhao et al. 2020).

Beyond structural modifications, heat treatment enhances digestibility and bioaccessibility, likely due to protein unfolding and the release of bioactive peptides. Mir et al. (2020) demonstrated an increase in albumin protein digestibility from 77% to 87.5%. Additionally, different heating methods such as conventional cooking, pressure cooking, and microwave processing altered the texture and viscoelastic properties of protein pastes, which is particularly relevant for applications in infant and senior nutrition (Gallego et al. 2021). Overall, these findings highlight the importance of heat treatment conditions to enhance protein functionality while maintaining desirable structural characteristics.

### 4.2 Ultrasonication

Ultrasonication (US) treatment is a method that decreases intermolecular aggregates, increases β‐conformation of protein, and increases protein solubility by inducing changes in the secondary structure of proteins (Akharume et al. 2021). This technology gained attention for its potential to reduce processing time and is often considered sustainable due to its reduced need for chemicals and water (Pojić et al. 2018).

US technology has been shown to modify the functional properties of plant proteins, by facilitating structural modifications that improve solubility, emulsification, and foaming capabilities. Research comparing various physical modification technologies (cold plasma, ball milling, superfine grinding, ultrasound; wet ball milling, and high‐pressure microjet) found that the US decreased walnut protein particle size and converted β‐sheets into α‐helices, enhancing solubility, foaming, and emulsification properties (Li et al. 2023). Gharibzahedi and Smith (2020) showed that US treatment (150 W for 30 min) improved the solubility (26.6%) and water‐holding capacity (38%) of chickpea protein by dissociating large protein aggregates into smaller proteins, thus enhancing protein‐water interaction. Further, US treatment (300 W for 20 min) enhanced chickpea gelling properties by 157.8% (Wang et al. 2020b), and improved emulsification and foaming capacities due to the exposure of hydrophobic groups (Kang et al. 2022). US treatment also shows promising results for hempseed protein, enhancing solubility and functionality through structural modifications (Yao et al. 2023).

Different US pretreatment mechanisms can also significantly impact protein structure. Ding et al. (2018) explored different US pretreatment mechanisms on grape seed protein. They found that mono‐frequency (20 kHz), simultaneous dual frequency (20/40 kHz), and alternate dual frequency (20/35 kHz) treatments enhanced the degree of hydrolysis and increased hydrophobic and total amino acids, demonstrating protein structure unfolding and hydrophobic exposure.

US treatment has been shown to significantly improve the digestibility and bioaccessibility of plant proteins, yielding promising results. Vanga, Wang, Orsat et al. (2020a) found that treating almond protein with US (20 kHz, 1–16 min) increased digestibility with longer treatment times, attributed to a reduction in β‐sheet structures and an increase in α‐helix structures. Wang et al. (2021a) reported that high‐intensity US treatment (20 kHz, 400 W, 50% duty cycle, 0–16 min) improved the digestibility of kiwifruit proteins from 35% to 62% after 16 min US treatment, although protein solubility decreased by 20%. Vanga, Wang and Raghavan (2020b) investigated the US application at the following conditions (25 kHz, 400 W, 1–16 min) to evaluate the relation between in vitro protein digestibility and structural alterations in soymilk proteins. The outcomes showed that the US enhanced digestibility at 16‐min treatment by increasing beta sheets in soymilk proteins. In a research work, the impact of ultrasound treatment on the bioavailability of commonly consumed dietary proteins (rice, oat, corn, and soy) was explored. The findings revealed that ultrasound significantly improved in vitro digestibility with enhancements of 9.49% for rice, 9.97% for oat, 8.19% for corn, and 9.84% for soy. Furthermore, bioavailability, assessed through a Caco‐2 cell model, was significantly increased following ultrasound pretreatment, with absorption rising by 5.59% for rice, 4.16% for oat, 8.78% for corn, and 18.69% for soy (Yuanqing et al. 2020). These studies showed the potential of the US for the improvement of plant protein digestibility.

The ability of ultrasound to fine‐tune texture while improving protein functionality offers an innovative approach for formulating foods with the right consistency for dysphagia patients. For instance, A study evaluated the application of US treatment (390 W, 20 min) on a specially designed commercial pea protein isolate to improve its texture for dysphagia‐friendly foods. The results demonstrated that ultrasound significantly enhanced the texture properties of pea protein isolate. The outcomes showed ideal characteristics for chewing and swallowing, including appropriate viscosity (33.31–704.2 mPa·s), softness (gel strength < 30 g), and wetness, with improved solubility and water‐holding capacity. Moreover, Yang et al. (2021) explored vegetarian meatloaves made from textured wheat protein under vacuum, US, and vacuum ultrasound treatments and compared their effects on texture and structure. Vacuum US treatment increased hardness by 176% and produced a smoother, tighter structure and more even moisture distribution, which had a texture closer to beef patties.

### 4.3 High‐Pressure Processing

High‐pressure processing (HPP) typically operates within the range of 200–700 MPa and significantly alters plant protein functionalities (Fidalgo et al. 2023). The effectiveness of HPP is influenced by process conditions such as duration, temperature, pressure, and the properties of the protein solution, including pH and ionic strength (Bolumar et al. 2021). High‐pressure treatment causes protein molecule rupture, denaturation, and aggregation by reducing solution volume (Zhang et al. 2023a). These factors collectively induce structural changes that impact protein functionality, particularly gelling properties.

HPP techniques significantly improve the physical and functional properties of plant proteins by modifying their structural characteristics. Ma et al. (2023) demonstrated that applying different homogenization pressures and cycles to chickpea protein improved surface hydrophobicity, reduced sulfhydryl content, and decreased particle size. These modifications enhanced solubility, foaming, and emulsification properties, resulting in more stable emulsions than untreated protein. Additionally, Alsalman and Ramaswamy (2021) applied varying pressure levels (227–573 MPa), treatment times (6–24 min), and chickpea protein powder concentrations (11%–29%). The results showed significant improvements in emulsion properties, with a decrease in protein aggregates to 33.3%, β‐sheets ranging from 4.2% to 87.6%, and α‐helix 50%, leading to enhanced protein digestibility.

HPP treatment can alter protein conformations and effects on digestibility. Linsberger‐Martin et al. (2013) demonstrated that applying 600 MPa at 60°C improved the digestibility of pea and bean proteins compared to heat treatment (100°C) and reduced antinutritional factor activity. (Hall and Moraru 2021) evaluated the impact of HPP (600 MPa/5°C/4 min) on the digestibility of lentil and fava bean protein concentrates. They found that processed samples exhibited higher gastric digestibility compared to untreated samples, with no negative effects on in vitro digestibility, suggesting HPP can be applied in new product formulations without adverse effects. Laguna et al. (2017) investigated HPP (600 MPa/5 min) on pea protein digestibility in apple and carrot purees at 37°C. Pea protein at pH 6.2 showed higher digestibility than at pH 3.6, likely due to the unfolding of globular protein subunits. Pea protein was more digestible in carrot puree compared to apple puree, attributed to procyanidin binding to pea protein. These findings offer insights into designing process parameters and selecting suitable food matrices for pea protein delivery.

HPP technology can improve food texture, which is crucial for developing products for individuals with altered deglutition (AD). Chickpea protein purées prepared with HPP (300–400 MPa for 3–9 min) achieved similar texture results to traditional texturizers (e.g., agar–agar or xanthan gum) without affecting the proximate composition (Fernández‐Pan et al. 2023). Peyrano et al. (2022) observed that cowpea protein, when treated at pressures ranging from 400 to 600 MPa and at concentrations greater than 10%, formed softer gels.

### 4.4 Pulsed Electric Field

Similar to HPP, pulsed electric field (PEF) processing has gained growing interest for its potential to enhance food proteins’ functional properties, which combines electrical, chemical, and thermal effects. Various studies have demonstrated the significance of process variables such as electrical frequency and electrical field strength on protein structural changes. These changes in protein structure variables can lead to alterations in denaturation and aggregation patterns, providing opportunities to improve protein functional properties (Pereira et al. 2024).

PEF processing can significantly alter plant protein structures. For instance, PEF treatment at 30–50 kV/cm can modify the protein secondary structure through the formation of hydrophobic and disulfide (S–S) bonds (Liu et al. 2011). Moderate electric field strength (150 V for 20 s at < 45°C) applied to sunflower proteins results in secondary and tertiary structural changes, causing bond breakage and amino acid chain cross‐linking (Subaşı et al. 2021). Similar outcomes have been observed in pea protein, where PEF treatment at moderate conditions (20 V/cm) leads to the unfolding of α‐helix into β‐sheets, increased surface hydrophobicity, and enhanced gelling characteristics (Y. Chen et al. 2022).

The effects of PEF processing on protein solubility vary depending on the specific conditions applied. For example, moderate PEF conditions (1.65 kV/cm, square pulse system) resulted in decreased solubility for peas (from 23.2% to 17.2%), rice (from 16.4% to 9.2%), and gluten (from 25% to 22.4%) concentrates due to protein unfolding and the formation of insoluble protein molecules (Melchior et al. 2020). Intermolecular interactions also contribute to decreased solubility. Conversely, PEF processing (35 kV/cm for 8 µs) increased the solubility of canola proteins (from 43.25% to 50.07%) and their water‐holding capacity, likely due to the exposure of hydrophobic groups on the protein surface (Zhang et al. 2017).

PEF processing can be combined with other techniques such as pH shifting and has been shown to significantly improve the functional properties of soy protein. Moderate PEF and pH 11 treatment increased solubility (SPI from 26.06% to 70.34%), enhanced surface hydrophobicity, flexibility, and free sulfhydryl content, which in turn improved emulsification and foaming characteristics (Wang et al. 2023b). These improvements are attributed to increased protein oxidation due to free radical production and protein conformation changes induced by polarization.

The water‐holding capacity and gelling properties of proteins are essential for determining the texture of food products, especially for enhancing palatability and ease of consumption in senior individuals. Water‐holding capacity of canola protein treated with PEF improved at a lower electric field strength of 25 kV/cm but reduced at a higher strength of 35 kV/cm (Zhang et al. 2017). In the case of pea protein isolate, applying lower electric field treatments resulted in gels that were more cohesive, elastic, and weaker, while also exhibiting enhanced water‐holding capacity (Chen et al. 2022). The variations in gelling properties observed across different studies may stem from differences in PEF parameters, including voltage, pulse wave shape, and the design of the treatment chamber. Guo et al. (2023) evaluated the effects of PEF and direct current electric fields (DCEF) on the water‐ and oil‐holding capacity, texture, and microstructure of wheat gluten. Both PEF and DCEF significantly impacted water‐holding and oil‐holding capacity, with DCEF having a stronger effect on textural properties. PEF resulted in smaller, uniform micropores, while DCEF produced a more compact structure.

### 4.5 Cold Plasma

Cold plasma applications on plant proteins are increasingly popular for improving physical and biochemical characteristics (Ji et al. 2022). Despite its potential, the effects of cold plasma on plant proteins are not comprehensively discussed.

Various researchers have analyzed cold plasma technology applications to enhance plant protein's functional properties. Zhang et al. (2021b) applied cold plasma to soy protein isolate dispersions, resulting in higher protein solubility than untreated samples. Plasma treatment generated reactive species that increased hydrophilic groups, improving water interaction with the protein. However, longer treatment times decreased solubility due to the overcrowding of protein micelles. Soy protein isolates treated at 120 Hz for 3 min showed a potential solubility increase of 282%, while Bußler et al. (2015) observed a 191% solubility increase under conditions of 8.8 kV for 10 min.

Various studies highlight the influence of cold plasma treatment on the structural changes in plant proteins and how these modifications can enhance their functional properties. For instance, research on soybean protein isolates treated at optimum conditions (18 kV for 15 min) led to alpha‐helices unfolding, followed by aggregation. The results indicated an increase in water‐holding capacity and emulsification activity (Sharafodin and Soltanizadeh 2022). In another research work, Mahdavian Mehr and Koocheki (2020) evaluated grass pea protein isolate under 9.4 and 18.6 kV conditions for 30 and 60 s. The protein absorption rate at the interface decreased at 9.4 kV and increased at 18.6 kV, with protein tertiary structure loss and increase in alpha and beta structures. These structural changes led to increased protein solubility and a decrease in emulsion droplet particle size. Protein physical properties depend on beta‐turns and beta‐sheets, with beta‐turns related to protein hydration and beta‐sheets responsible for protein–protein interactions. The possibility of protein aggregation decreased with a reduction in beta‐sheets (Held et al. 2019). Ji et al. (2018b) observed higher water‐holding capacity in peanut protein linked to decreased beta‐sheets. Misra et al. (2015) reported similar outcomes for wheat flour subjected to cold plasma, with decreases in beta‐sheets and increases in alpha‐helices and beta‐turns. Yu et al. (2020) applied cold plasma treatment (5 kV; 40 kHz) for varying durations (0–240 s) on flaxseed protein. The results showed that conformational changes increased surface hydrophobicity and disulfide bonds with longer treatment durations.

Cold plasma treated proteins are proving to be effective in enhancing the bioaccessibility and controlled release of bioactive compounds in delivery systems. Yu et al. (2021) developed a glycoprotein emulsion using cold plasma treated denatured pea protein isolate combined with sesbania gum for β‐carotene encapsulation. The emulsion achieved an encapsulation efficiency of over 97% and showed improved stability when exposed to heat and salt. Additionally, the glycoprotein‐stabilized emulsion demonstrated greater bioaccessibility and a more sustained release of β‐carotene. Cold plasma can significantly enhance the gelling properties of plant proteins, which is essential for improving the texture of foods tailored for senior consumers. For example, cold plasma as a novel nonthermal method was explored to improve the gelling properties of pea protein concentrate. Native pea protein concentrate (12 wt%) did not form a gel at 90°C, while cold plasma‐treated pea protein concentrate exhibited good gelling capabilities at 70–90°C, resulting in gels with a three‐dimensional network structure, high mechanical strength, viscoelasticity, and excellent water‐holding capacity (Zhang et al. 2021c). With its short exposure time and energy efficiency, cold plasma has significant potential for enhancing plant protein as a gelling agent in plant‐based foods, including meat alternatives and egg substitutes. Rahman and Lamsal (2023) examined the effects of cold plasma on the gelling properties of mung bean protein dispersions. CP treatment at 80 kV for 5 min reduced the minimum gelling concentration from 16% to 14% w/v and lowered the gelling temperature to 65°C compared to the control (75°C). Additionally, cold plasma‐treated dispersions had a six‐fold higher storage modulus and significantly increased hydrophobic bond formation, enhancing the firmness and gel structure of mung bean protein. Cold plasma processing may introduce trade‐offs, including lipid/protein oxidation and potential flavor changes; therefore, optimization of processing conditions is essential, particularly for products designed for senior populations where sensory acceptance is critical.

### 4.6 Extrusion

Extrusion is a physical modification approach suitable for the industry. This method involves high temperature, pressure, and shear in an extruder, which unfolds, linearizes, and recrosslinks the protein structure. This weakens binding forces changing the protein molecules from spherical aggregation to fibrous structures and inducing denaturation (Samard and Ryu 2019). This is usually used as a commercial technique for transforming plant protein materials into textured vegetable proteins (Dekkers et al. 2018), and be used to improve the functionality of plant proteins for encapsulation and nutrient delivery. Extrusion parameters such as screw rotation speed and feed moisture content can alter end‐product properties (Zhang et al. 2019).

High‐moisture extrusion is a potential technology for processing plant‐based protein products into desirable textures and mouthfeel (Sui et al. 2024). Extrusion affects plant proteins’ rheological properties, thereby influencing the final extrudates. For example, extrusion applied to rice proteins at varying conditions (screw speeds of 100–250 rpm, temperatures of 90–150°C, and moisture contents of 25%–40%) resulted in improved protein solubility (45.23%), water‐holding capacity (37.74%), and emulsion stability (152.82%). During extrusion, sulfhydryl, disulfide, ionic bonds, and hydrophobic interactions decreased, except under specific conditions (200 rpm, 130°C, and 40% moisture), while hydrogen bond content increased. Protein microstructure was altered, producing protein aggregates with a tight structure, and the α‐helix content decreased (Gao, Sun et al. 2022b).

Emerging plant‐based meat analogues greatly benefit from extrusion technology, as it enhances their structural and functional properties, making them more suitable for diverse food applications. Pea protein isolate subjected to extrusion at various conditions (90–130°C and 65%–70% moisture) showed structural changes from a spherical to a fibrous network, with broken secondary and tertiary structures forming new cross‐links. Extrusion at high temperatures and suitable moisture content caused partial unfolding and realignment of protein structures (Sun et al. 2022b). Extrusion reduced α‐helix/β‐sheet values and free amino acid content, improving protein digestion and absorption (Y. Wang et al. 2024e).

Extrusion technology enhances starch and protein digestibility but can also convert nutrients into non‐nutritious compounds. For instance, the extrusion of various pulses (Amarillo peas, Dun peas, lentils, chickpeas, and faba beans) with soybean meal (control) at different moisture levels (18% or 22%) and temperatures (110, 130, or 150°C) showed enhanced crude protein content in Amarillo, Dun peas, and lentils, but a decline in soybean meal. Methionine content increased in chickpeas and lentils, while cysteine content increased in Amarillo peas but decreased in soybean meal. These results indicate that extrusion positively affects protein and amino acid content, though the changes vary by protein type (Cargo‐Froom et al. 2023). Extrusion increased soybean protein digestibility from 78.5% to 88.9% by inducing secondary structure changes (de la Rosa‐Millán et al. 2019). It also impacts wheat protein, increasing nitrogen content in digesta (Lin et al. 2022). Extrusion can efficiently reduce antinutritional factors and enhance protein digestibility. For example, extrusion applied to pea, fava, quinoa, hemp, and oat concentrates produced pulse‐rich, cereal‐rich snacks with a 71% reduction in trypsin inhibitor activity and enhanced in vitro protein digestibility due to protein aggregation and enzyme activity reduction (Duque‐Estrada et al. 2023).

Additionally, understanding the influence of extrusion parameters is essential for tailoring protein properties to meet specific functional and textural requirements in food products. Hempseed protein extruded at feed moisture content (30%–60%) and screw rotation speed (200–400 rpm) showed that lower feed moisture and higher rotation speed resulted in higher texturization and expansion index while density and color values decreased. These outcomes are crucial for understanding extruded protein characteristics important for industrial applications (Rajendra et al. 2023). The results of extrusion technology on plant proteins are promising for enhancing their functionality, making them more suitable for senior nutrition.

### 4.7 Irradiation

Gamma irradiation and electron beam irradiation (EBI) are nonthermal technologies that extend the shelf life of food products and induce changes in protein structures. These technologies generate hydroxyl and superoxide anion radicals, leading to alterations in primary, secondary, tertiary, and quaternary protein structures (Han et al. 2018).

Gamma irradiation can significantly change protein structure through cross‐linking and fragmentation. For instance, gamma irradiation altered rice protein's secondary and tertiary structures, as demonstrated through UV–VIS spectra and luminescence measurements. pH measurements confirmed chemical composition changes in tryptophan and tyrosine amino acids in irradiated samples (Baccaro et al. 2018). For sunflower protein isolate, gamma irradiation caused changes in the α‐helix and β‐sheet contents, leading to increased thermal stability, surface hydrophobicity, emulsifying, foaming, and oil‐holding capacities. However, the water binding capacity decreased (Malik et al. 2017). The emulsification properties of sesame protein improved after gamma irradiation (Meinlschmidt et al. 2016), and increased the digestibility of sesame protein (Hassan et al. 2018).

EBI involves high‐energy electrons that impact the chemical and molecular configuration of proteins, leading to unfolding and denaturation, which enhances protein functionalities. For example, EBI caused structural changes in wheat germ protein hydrolysates, decreased molecular weight and surface hydrophobicity, and improved emulsification and foaming characteristics. The antioxidant ability also increased with EBI exposure (Wang et al. 2019; X. Zhang et al. 2020c). EBI enhanced the antioxidant ability and emulsification activity in rice protein (from 145% to 204%) due to protein aggregation and cross‐linking (T. Li et al. 2019b). EBI exposure improved the digestibility of soy protein and reduced trypsin inhibitor content (Kumar et al. 2017). EBI‐assisted enzymatic hydrolysis on rice proteins enhanced solubility and emulsification properties (R. Li et al. 2019a; Zhang et al. 2020b).

The effects of irradiation on protein structure and functionality are complex and dose dependent. In watermelon seed kernel protein, irradiation reduced β‐sheet content and increased random coil structure, particle size, and surface hydrophobicity, increasing solubility and digestibility (Z. Li et al. 2020c). For chickpea protein concentrate, protein solubility increased at 10 kGy and 15 kGy, while it reduced at 20kGy. The structural changes impacted the physicochemical properties (Y. Zhang et al. 2023b). In addition, El‐Niely (2007) revealed that irradiation at 5, 7.5, and 10 kGy on peas, cowpeas, lentils, kidney beans, and chickpeas reduced antinutrients phytic acid and tannins while lowering available lysine (AL). In vitro protein digestibility and protein efficiency ratio improved in a dose‐dependent manner compared to nonirradiated samples.

EBI has notable effects on the texture by increasing the water‐holding capacity of proteins. Yao et al. (2022) investigated the effects of different γ‐irradiation doses (0, 0.5, 1, 2, 3, 5 kGy) on the physicochemical and functional properties of rice protein. At 2 kGy, rice protein showed the smallest particle size, highest zeta potential (33.58 mV), least β‐sheet content (31.16 ± 0.16), and the highest random coil content (14.56 ± 0.06). The microstructure became rough with deep pore depressions, and surface hydrophobicity peaked at 160.45 ± 2.98. Functional properties also improved, with the highest solubility (69.18 ± 1.07%), water (5.89 ± 0.08 g/g), and oil (3.45 ± 0.04 g/g) holding capacities, and enhanced emulsification. The outcomes demonstrated the textural properties were enhanced, as the increased water‐holding capacity contributed to a firmer and more cohesive protein structure.

## 5 Chemical Modifications

Chemical modification is a widely used and effective technique for protein functionality improvement. This processing method makes it easier to target the specific properties of plant proteins by their structure and function alterations. These modifications may take advantage of amino acid side chains to obtain desired characteristics (Feng et al. 2021). In contrast, physical methods typically induce general changes in protein properties, often lacking precision in achieving specific functional enhancements.

**FIGURE 3:**
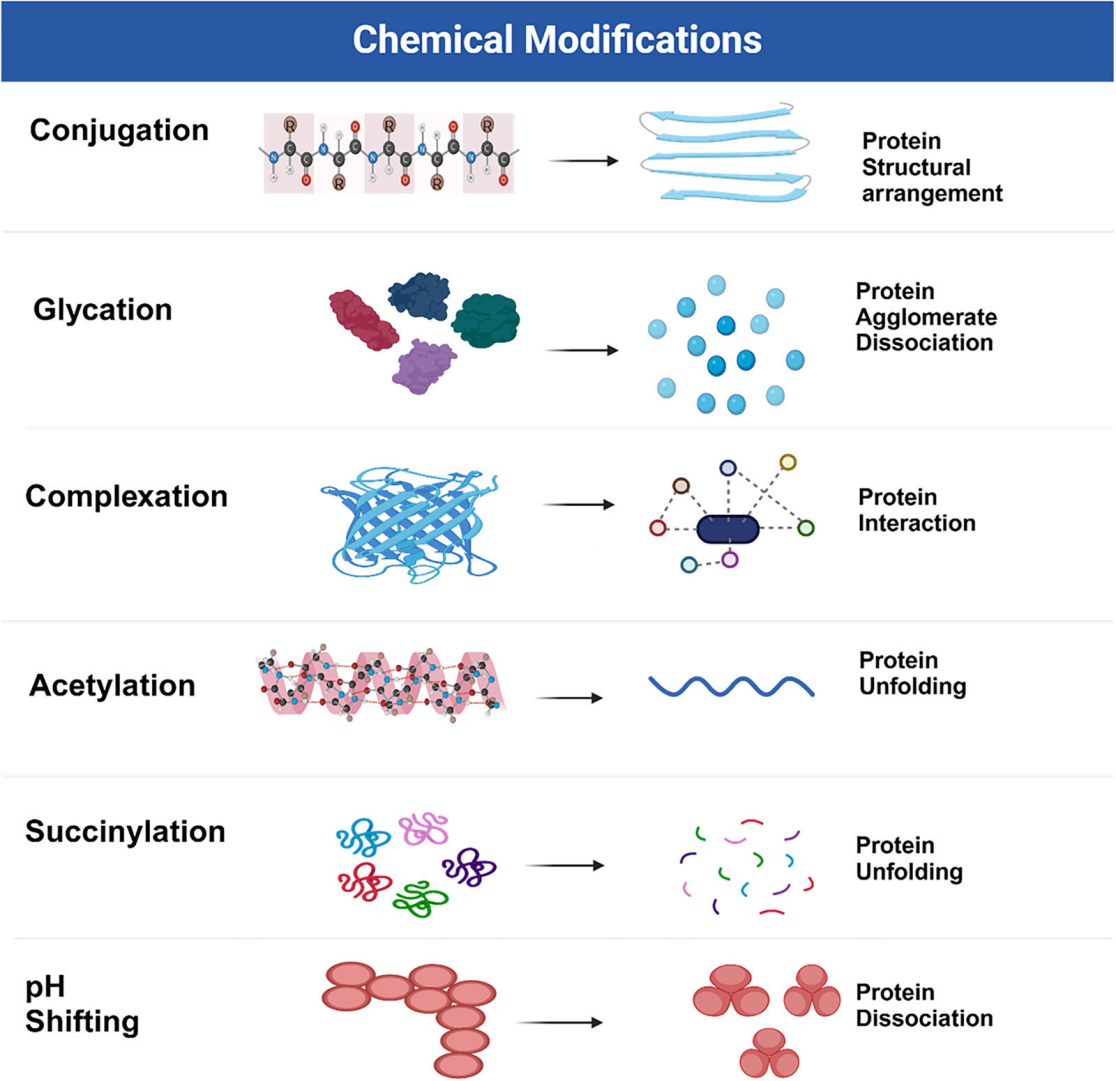
Illustration of chemical modification processes for plant proteins (created in BioRender).

Figure 3 illustrates various chemical modification techniques applied to plant proteins, including glycation, acetylation, conjugation, pH shifting, and complexation. Each method induces specific structural changes such as protein aggregation, unfolding, binding, agglomerate dissociation, and molecular interactions. These modifications alter the protein's functionality, enhancing its properties for various applications. Table 2 summarizes the impact of chemical modification methods on plant protein functionality. Techniques such as glycation, conjugation, and pH shifting significantly enhance solubility, emulsifying properties, and foaming capacities in proteins like soy, pea, and hemp. Combining pH shifting with ultrasound has been shown to increase solubility up to 99%, while acetylation and succinylation improve water‐ and oil‐holding capacities across different plant protein.

**TABLE 2:** Impact of chemical modification methods on plant–protein functionality improvement.

Modification approach	Treatment conditions	Protein type	Physical properties	Functional properties	Molecular structure	Biological properties	References
**Conjugation**	Soy: Gum Arabic (1:1), ultrasonic 30 min (180 W), wet heating (95°C for 1–6 h), Dry heating (60°C and 79% humidity)	Soy	–	↑ Degree of grafting; Dry heat (53.61%), Wet heat (28.07%) ↑ Viscosity for dry heat, ↓ Viscosity for wet heat ↑ Encapsulation efficiency ↑ Storage stability ↓ Interfacial tension	–	–	Feng et al. (2023)
	Dry heat (60°C for 1 day), Wet heat (95°C for 90 min)	Soy	—	↓ Surface hydrophobicity,	—	↓ Free amino group Content (0.77–0.62 mmol/g)	Feng et al. (2021)
	Heating (80°C for 2 h), Centrifugation (8000 × *g*, 20 min)	Pea	↓ Particle size (157.53 nm to 128.63 nm)	↑ Emulsification efficiency	—	—	C. Sun et al. (2022a)
	Humidity (49.0%) and 60°C for 24 h	Pea	↑ Solubility (90%)	↓ Surface hydrophobicity	—	↑ Digestibility (100%)	Schneider et al. (2023)
	Hemp: Xylose (1:2, w/w), 70°C for 24 h	Hemp	↑ Solubility (+3.6‐fold)	↑ Emulsion stability (+15‐fold) ↑ Foaming stability (+1.7‐fold)	—	—	Karabulut and Feng (2024)
	pH (9.0), (23 ± 2°C)	Lentil	↓ Turbidity ↑ Solubility	↓ Surface hydrophobicity ↑ Radical scavenging and reducing power ↓ Interface tension	↓ β‐sheet,	—	Parolia et al. (2022)
	Grass pea protein; Xanthan gum (20:1), 80°C, (1, 6, 12 h)	Grass Pea	↑ Solubility (98.88%)	↑ Degree of glycation (up to 37.43%) ↑ Foaming capacity (by 45.17%) ↑ Foaming stability (by 37.17%),	—	—	Amiratashani et al. (2024)
	pH (9.0), Ultrasound (400 W for 20 min)	Hemp	↑ Solubility ↓ Particle size	↑ Emulsifying ability ↑ Surface hydrophobicity ↑ Zeta‐potential	—	—	Liu et al. (2023)
	HPI and EGCG (1:1), pH (9.0), Stirring (24 h), 25°C	Hemp	↓ Particle size (to 100 µm) ↑ Solubility (from 39.4% to 50.6%)	↑ Emulsion activity ↑ Emulsion stability	—	—	Pang et al. (2021)
**Complexation**	pH (3.2), Oak protein isolate: gum Arabic (4:1)	Oak	↑ Solubility	↑ Surface hydrophobicity ↑ Water‐holding capacity ↑ Oil‐holding capacity ↑ Foaming ability ↑ Emulsifying ability	—	—	Naderi et al. (2020)
	Pea protein: Tragacanth gum (2:1), pH (4.5)	Pea	↑ Solubility (from 2.5% to 14.6%)	↑ Zeta potential (−28,5 mV)	—	—	Carpentier et al. (2021)
	Potato protein: Gellan gum (20:8) wt%, Incubation 20 min, Stirring 5 min, pH 3–8	Potato	—	↑ Zeta Potential	↑ Microstructural uniformity	—	Hu et al. (2024)
	Hemp seed: Gum Arabic (0.5:1, 1:1, 2:1, 4:1, 6:1, 10:1, and 13:1 (*w*/w)), pH (7.0–2.0)	Hemp	↓ Particle size	↑ Zeta Potential (−21 mV to 18 mV)	—	—	Plati et al. (2021)
	Acetic anhydride (AA) or succinic anhydride (SA) at 0.3 or 0.6 g	Pea	—	↑ Oil‐holding capacity (up to 2.20 ± 0.05) ↑ Water‐holding capacity (up to 7.01 ± 0.31 g) ↑ Gelling ability ↑ Emulsion capacity ↑ Emulsion stability	—	↓ Digestibility	Shen and Li (2021)
	pH 8, 0.4 mL of acetic anhydride/g	Rice	↓ Solubility	↑ Emulsifying ability (1.83%–14.74%) ↑ Water‐holding capacity (4.89–5.62 g/g) ↓ Foaming capacity	—	—	Miedzianka et al. (2023)
	Acetic anhydride (0.02, 0.06, 0.10, 0.14, and 0.20 g/g of protein)	Sesame	↓ Solubility (from 43% to 31%)	↓ Water‐holding capacity ↑ Emulsifying ability ↑ Emulsion stability ↑ Foaming Ability (124%–160%)	—	—	Castillo‐Ortega et al. (2024)
	Succinic anhydride (10%, 20%, 30%, 40%, and 50%)	Rice	↑ Solubility (4%–36%) ↓ Particle size (from 340.37 to 112.5 nm)	↓ Surface hydrophobicity (by sevenfold) ↑ Water‐holding capacity (by seven times) ↑ Oil‐holding capacity (by 3–7 times) ↑ Foaming capacity (from 9.12% to 102.44%) ↑ Emulsifying activity	—	—	Luo et al. (2024)
	Succinic anhydride (10% (v/v)	Black bean	↓ Particle size ↓ Turbidity	↓ Zeta potential ↓ Surface hydrophobicity ↑ Water‐holding capacity (up to 65%) ↑ Hydrogel stability	—	—	Cheng et al. (2024)
	Glutenin; Succinic anhydride (1:1, v/v), 600 rpm, 2–4 h	Walnut	↓ Particle size (by 122‐fold)	↓ Zeta potential (by 0.27‐fold) ↑ Emulsifying ability ↑ Gelling ability	—	—	Yuanli Wang et al. (2024d)
**pH shifting**	pH 2 or 10, with and without heat (80°C)	Pea, rice, oat, and hemp	↑ Solubility	↑ Water absorption capacity	—	—	Tang et al. (2023)
	pH (7, 8, 8.5, 9, 10, 11, and 12)	Alfalfa	↑ Solubility at pH 11 (from 50% to 80%)	↑ Foaming stability (> 60%) ↑ Gelation ability	—	—	Nissen et al. (2021)
**pH shifting + High pressure**	Acidic (pH 2–6) or alkaline (pH 8–12) at 250 MPa	Quinoa	↓ Particle size (54 nm) ↑ Solubility (78.79%)	↑ Surface hydrophobicity	—	—	Yildiz and Yıldız (2023)
**pH shifting + Ultrasound**	pH 7, US power (200, 300, 400, 500, and 600 W)	Pea	↑ Solubility (82.42%)	—	↑ β‐Sheet content	—	Zhang et al. (2022a)
	20 kHz, 400 W, 20 min; pH 10 and 12)	Pea	↑ Solubility (99%)	—	—	—	Yang et al. (2022a)

Chemical conjugation including glycation through the Maillard reaction is an effective chemical modification technique for improving plant protein functionality (Zheng et al. 2022). It reduces protein aggregation due to steric hindrance from covalent binding. It also significantly enhances the encapsulation efficiency and storage stability by lowering interface tension and increasing electronegativity (Feng et al. 2023). For instance, X. Sun et al. (2022c) demonstrated conjugates of gum acacia with plum seed protein isolate enhanced emulsion stability and emulsifying capabilities, reduced flocculation, and improved overall solubility.

The combination of conjugation methods with physical treatments, such as PEF, can greatly enhance the amino acid content. Sun et al. (2022b) demonstrated that PEF treatment significantly impacts Maillard reaction conjugates by improving their solubility and emulsion stability. This combined approach also increases the concentration of free amino acids and strengthens conjugation, effectively preventing heat‐induced protein aggregation.

Chemical modification approaches such as glycation and acylation play a key role in improving the textural properties of plant proteins, enhancing their functionality for various food applications. Z. Wang et al. (2018b) explored acylation and glycation to enhance the gelation properties of rapeseed protein isolates. Both processes increased surface hydrophobicity, gelation, water‐holding capacity, and texture, with reduced least gelation concentration and surface roughness.

Complexation (acetylation, succinylation) starts with covalent bonding between amino groups of protein and carboxyl groups of reducing sugars producing Schiff bases and Amadori compounds. This process can enhance functional properties by deliberately forming covalent bonds between amine and carbonyl groups (Higa and Nickerson 2023). A recent research work investigated the development of plant‐based meat analogs using coacervation and thermal gelation of gellan gum and potato protein. By adjusting pH to modulate electrostatic interactions, researchers controlled the structural organization and texture of the biopolymer composites. Findings showed that pH significantly influenced the charge dynamics, air bubble formation, and shear modulus, enabling the creation of meat‐like textures with varying fibrous structures and gel strengths (Hu et al. 2024). Kannamangalam Vijayan et al. (2021) evaluated the complexation of curcumin with pea protein isolate and whey protein isolate. Thermodynamic analysis indicated a hydrophobic interaction. The whey–curcumin complex demonstrated better solubility at pH 3, 5, and 7, while the pea‐curcumin complex excelled at 80°C, with solubility values of 1.16 mg/g and 1.02 mg/g, respectively, at pH 7. Both protein complexes showed a similar score for the bioaccessibility of curcumin.

Acetylation involves the interaction of acetyl groups with the amine groups of proteins. This modification could unfold proteins and improve protein solubility by exposing hydrophilic groups and increasing hydrophobicity (Heredia‐Leza et al. 2022). However, solubility may decrease at pH ranges of 2–7 due to hydrophobic linkages forming in alkyl and aromatic amino acid groups, leading to protein aggregation (Jaworska et al. 2020). Acetylation has been shown to improve the emulsification capacity of chickpea protein by 19%, due to the formation of lipophobic and hydrophobic residues (Heredia‐Leza et al. 2022). It also enhances foaming ability of pea protein by reducing the positive charges of amino groups, which decreases molecular size and improves water and air interaction (Shen and Li 2021).

Succinylation involves the modification of proteins with succinyl groups. Dnyaneshwar Patil et al. (2024) explored succinylation on functional properties of chickpea protein. The outcomes revealed improved water‐holding capacity (39.83%), oil‐holding capacity (54.02%), and solubility (7.20%). These improvements are attributed to changes in the protein's surface and structure. Cheng et al. (2024) also investigated the effect of succinylation on black bean proteins and their gel properties. Succinic anhydride at 10% (v/v) resulted in 92.53% acylation, reduced particle size, and improved protein flexibility as indicated by dissociation of secondary structures. The hydrogel formed a stable three‐dimensional network with enhanced elasticity and strength after protein succinylation.

The pH shifting treatment involves pH alterations toward an acidic or basic environment followed by neutral conditions. Protein unfolding and refolding enhance the functional properties. It has been proven an efficient way to enhance the plant protein's functionalities, including soy protein (Jiang et al. 2018), pea protein (Jiang et al. 2017), and hemp seed protein (Q. Wang et al. 2018a). Liu et al. (2015) investigated the effects of extreme acid pH‐shifting and mild heating on soy protein isolate. Incubation at pH 1.5 for 5 h and subsequent heating (50 or 60°C) led to increased gel penetration force, and higher hydrophobicity. The results showed that combining pH‐shifting with heating enhances plant protein functionality by promoting structural alterations.

From a food‐industry perspective, the feasibility of chemical modification approaches depends on processing scalability, regulatory approval, cost, and consumer acceptance, particularly for senior‐adult foods. Widely used methods, such as limited oxidation or cross‐linking, are compatible when appropriately controlled. However, excessive chemical modification may raise regulatory and consumer concerns related to clean‐label expectations and perceived “overprocessing,” especially in senior nutrition products. Importantly, harsh chemical treatments can alter amino acid availability through side‐chain modification or reduced digestibility, potentially compromising protein quality for senior adults with higher anabolic requirements. Therefore, mild and well‐controlled chemical approaches that enhance functionality without adversely affecting nutritional value or sensory quality are the most realistic and acceptable strategies for foods targeting aging populations.

## 6 Enzymatic Modifications

Enzymatic modification is an effective, sensitive, and cost‐efficient method for modifying plant proteins (Barać et al. 2011). This approach enhances the application of plant proteins in the food industry by improving their digestibility, and functional properties, and may also reduce allergenicity. Additionally, enzymatic modification yields products with various potential health benefits, including anticancer, antioxidative, and immunomodulatory effects (Aondona et al. 2021; Görgüç et al. 2020).

This modification approach begins with fermentation, where microbial activities break down macromolecules into smaller, more easily digestible and absorbable molecules (Campbell‐Platt 1994). Fermentation can be spontaneous, involving Indigenous microorganisms, or nonspontaneous, using a starter culture (Fadimu et al. 2022). In the context of plant proteins, fermenting organisms such as bacteria, yeast, and molds produce proteolytic enzymes that convert proteins into peptides and amino acids of various molecular sizes. For instance, Shi et al. (2024) investigated lentil protein with fermentation treatment using *Aspergillus niger*, *Aspergillus oryzae*, and *Lactobacillus plantarum*. The results demonstrated increased solubility, foaming capacity, and oil‐holding capacity. Brückner‐Gühmann et al. (2019) characterized lactic acid fermentation on oat‐based gels. The outcomes showed improved water‐holding, oil‐holding, and textural properties. Fermentation also has potential in plant protein digestibility and bioaccessibility improvement. For instance, fermentation of soybean protein isolates with *Bacillus subtilis* enhanced the in vitro digestibility 2.43‐fold and also increased the bioaccessibility of amino acids (Ketnawa and Ogawa 2021).

Enzymatic hydrolysis is another biological method for modifying plant proteins that involves adding proteolytic enzymes to break peptide bonds, converting them into low molecular weight, and cross‐linked peptide chains (Eckert et al. 2019). This process affects the amino acid sequence, digestibility, and functional characteristics of plant proteins (Akharume et al. 2021). Enzymatic hydrolysis of walnut protein using papain reduced molecular weight, increased flexibility, and improved emulsifying ability, demonstrating the approach's effectiveness (Feng et al. 2024). Klost et al. (2020) investigated interactions within pea protein and pea protein hydrolysate gel networks, focusing on their rheological properties. Findings revealed that hydrophobic interactions, primarily involving the legumin fraction, dominated, with electrostatic interactions between vicilin and legumin‐β also playing a role. Enzymatic hydrolysis, particularly with trypsin, increased vicilin's involvement in the gel structure by enhancing legumin‐β availability.

**FIGURE 4:**
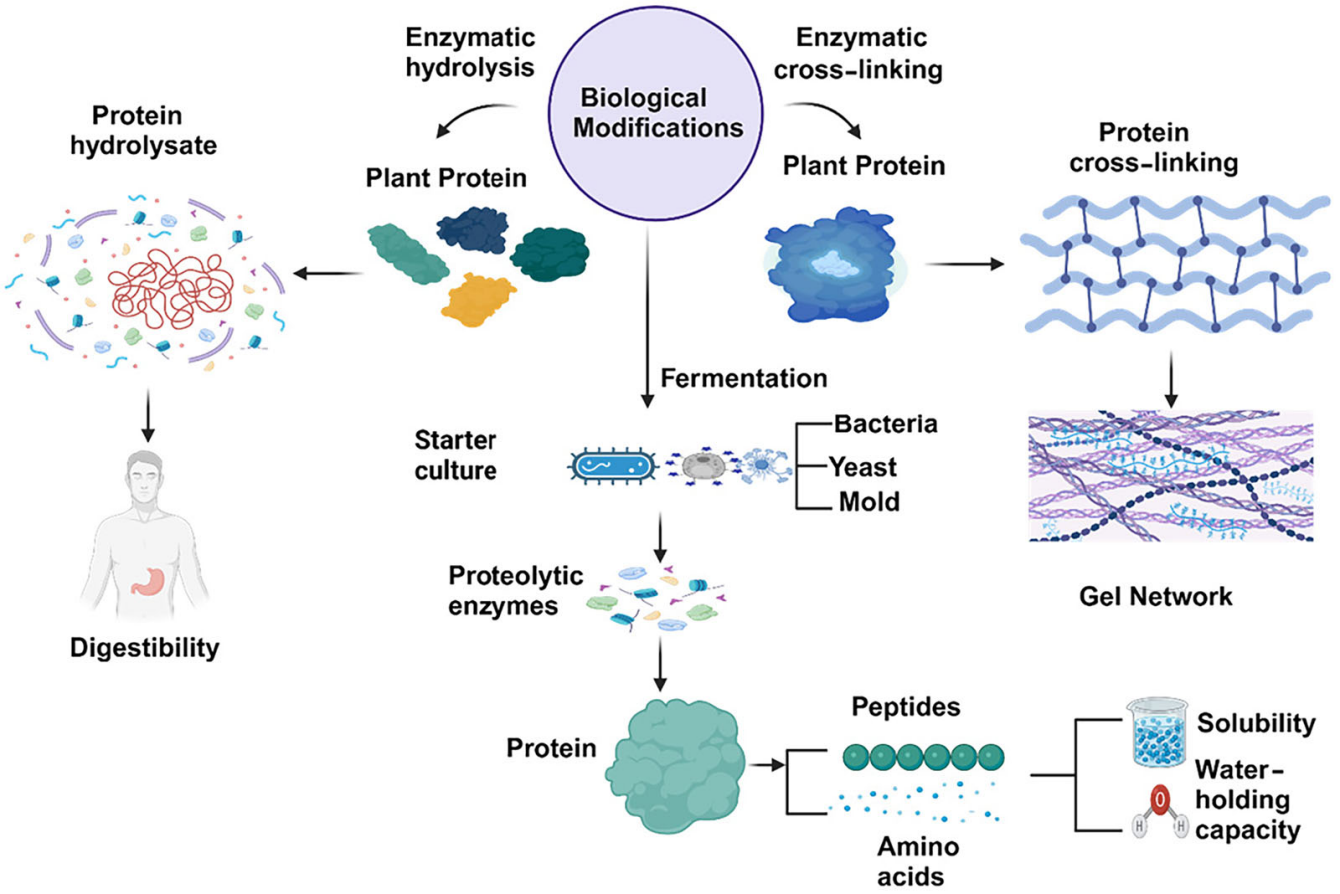
Biological modification approaches for plant proteins including fermentation, enzymatic hydrolysis, and cross‐linking processes (created in BioRender).

Figure 4 depicts the processes involved in the biological modification of proteins. Enzymatic hydrolysis breaks down proteins into smaller peptides, resulting in enzymatic hydrolysates. Enzymatic cross‐linking facilitates the formation of a gel network through interactions between acyl donors and acceptors. Fermentation employs starter cultures, including bacteria, molds, and yeast, to produce proteolytic enzymes, which hydrolyze proteins into bioactive peptides and amino acids. These modifications enhance the solubility, foaming capacity, and overall functional properties of the proteins.

In addition to protein hydrolysis, enzymatic cross‐linking is another effective approach. This method uses enzymes such as transglutaminases (TG), peroxidases, laccases, sulfhydryl oxidases, and tyrosinase to cross‐link or polymerize proteins (Buchert et al. 2010). This approach is also utilized to enhance the functionality of plant proteins. Yi et al. (2024) demonstrated the effect of laccase‐catalyzed cross‐linking on pea protein structural, emulsifying, and gelling properties. The outcomes showed enhancement in emulsifying activity, emulsifying capacity, gel strength, and water‐holding capacity.

Table 3 highlights the impact of various enzymatic treatments on the functional and physical properties of different protein sources. Enzymes like Flavorzyme, Pepsin, and Alcalase significantly improved solubility, emulsifying capacity, and foaming capacity in proteins from rice, faba beans, peas, and other sources. We have summarized the effectiveness of these treatments depended on specific conditions, such as enzyme/substrate ratios and pH levels, demonstrating that enzymatic hydrolysis can enhance the functionality of plant‐based proteins for food applications.

**TABLE 3:** Impact of enzymes treatment on plant protein functionality.

Enzymes	Conditions	Protein type	Physical properties	Functional properties	References
**Flavorzyme**	Enzyme: substrate ratio of 2 g/100 g protein	Rice	↑Emulsion droplet size	↑ Antioxidant capacity up to (2.5‐fold) ↑ Emulsifying capacity (10‐fold) ↑ Encapsulation efficiency (from 80.2% to 89.5%)	Gomes and Kurozawa (2020)
**Pepsin**	Enzyme/substrate ratio 5/100 (w/w)	Faba	↑Solubility (from 24.4% to 88.8%)	↑Foaming capacity (from 31.2% to 122.2%) ↑Oil‐holding capacity (from 6.12 to 8.21 g/g)	Eckert et al. (2019)
**Trypsin**	Hydrolysis times (15, 30, 60, and 120 min), pH 4.5	Pea	—	↑Foaming capacity (2271%) ↑Emulsifying capacity (719 mL/g)	García Arteaga et al. (2020)
**Pepsin, Trypsin, Protamex**	Pepsin (pH 2.0 and 37°C), trypsin (pH 7.0 and 37°C), and protamex (pH 7.0 and 50°C), (30, 60, 90, and 120 min)	Sea buckthorn	↑Solubility ↓Particle size (from 790 to 295 nm),	↑ Zeta potential ↑ Emulsifying ability ↑ Surface hydrophobicity,	Khan et al. (2023)
**Endoproteases**	Enzyme dosages (0.2, 2, and 20 U/g of substrate protein)	Rice	↑Solubility (from 1.0% to 51.2%)	↑ Zeta potential ↑ Surface hydrophobicity ↑ Foaming capacity (up to 500%) ↓ Gelling ability ↑ Water‐holding capacity (to 3.4 g/g)	Nisov et al. (2020)
	Peanut powder (9.7, 9.4, 9.1, 8.8, and 8.5 g), Tween 20 (0.3%, 0.6%, 0.9%, 1.2%, and 1.5%)	Peanut	↓ Solubility	↑ Emulsifying ability ↓ Surface hydrophobicity ↑ Gel strength	S. B. Zhang et al. (2020a)
**Proteolytic enzymes**	Alcalase 2.4 L, Enzyme/substrate% (0.5), (50°C, pH (8.0)	Lupin	↑ Solubility (to 75%)	↑ Foaming capacity (from 980% to 3614%) ↑ Emulsifying ability (to 679 mL/g)	Schlegel et al. (2019)
**Alkalize hydrolysis and Transglutaminase**	Degrees of hydrolysis (2.5%–10.0%), pH 8.0, (Enzyme/substrate) of 0.25%, 0.5%, 1.0%, 2.0%, and 3.0% (w/w)	Soy	↑ Solubility	↑ Emulsion activity ↑ Emulsion stability ↑ Foaming capacity (by 8.3%–28.5%), ↑ Foaming stability (by13.9%–176.5%)	Q. Zhang et al. (2021a)
**Endogenous enzymes and savinase**	Enzyme/protein ratio 1:1 (U/mg), pH (9.0, 55°C)	Black cumin	↑ Solubility	↑ Emulsifying ability ↑ Emulsion stability ↓ Foaming capacity ↓ Foaming stability	Trigui et al. (2021)
**Enzymatic hydrolysis**	Degree of Hydrolysis (5%, 10%, 15%, and 20%), pH 7.8	Chickpea and lentil	↑ Solubility chickpea (from 2% to 10%); lentil (from 2% to 31%)	↑Surface hydrophobicity (26 mA U), ↓Water‐holding capacity Chickpea (from 3.04 to 2.72–2.80 g/g Lentil (3.53–3.30 g/g) ↑Oil‐holding capacity chickpea (from 1.11 to 2.02 g/g); Lentil (from 1.12 to 2.46 g/g)	Thirulogasundar et al. (2024)

### 6.1 Plant Proteins in Nutrient Encapsulation and Delivery for Elder Nutrition

The development of nutrient and bioactive delivery carriers has become an attractive field due to the growing global demand for nutritious and healthy food. Food proteins are particularly valued for their ability to create nutrient‐delivery vehicles, offering high biodegradability, biocompatibility, and potential for structural and functional modification (Amagliani and Schmitt 2017; Sanidad et al. 2019). These proteins can form various configurations, including spherical nanoparticles, hollow particles, gels, and fibrils, which provide diverse mechanisms for stability and digestion profiles in nutrient‐delivery systems (Yan et al. 2020).

Furthermore, food proteins’ physicochemical and functional properties are highly tunable through heating, mechanical forces, and chemical techniques. Plant proteins such as soy proteins, zein, and wheat gliadins are used in delivery vehicles like micro and nanoparticles, fibers, films, and hydrogels, which are more economical compared to animal proteins. For example, vegetable protein‐based emulsion systems can be applied to deliver bioactive compounds. Zein and soy proteins are often used to develop nanofibers for controlled nutrient release, such as gallic acid (Moomand and Lim 2014) and β‐carotene (Neo et al. 2013). Xu et al. (2020) successfully encapsulated and slowly released lutein using rice protein combined with carboxymethyl cellulose. Kim and Peterson (2021) developed zein nanoparticles through liquid‐liquid dispersion to encapsulate menthol, achieving an encapsulation efficiency of over 90%. Reboredo et al. (2022) created zein nanoparticle‐coated poly(anhydride)–thiamine for insulin encapsulation, showing better release effects than free insulin.

Bean protein, known for its good biocompatibility and bioaccessibility, is another promising material for bioactive substance delivery. Zhang et al. (2021d) developed a curcumin–soybean protein nanocomposite with a particle size of 244.7–344.7 nm, achieving optimal storage stability and curcumin loading. Emulsion gels present a novel oral delivery carrier capable of loading both hydrophilic and lipophilic components. M. Zhang et al. (2022b) developed soy protein isolate‐sugar beet pectin emulsion gels using laccase and TG, which showed high density, lower digestion, and controlled release of coloaded β‐carotene and riboflavin. These findings demonstrate the potential of plant‐based emulsion gels for encapsulating and controlling the release of bioactive compounds.

Proteins are amphiphilic molecules that adsorb at oil–water interfaces, forming protective films around oil droplets, a property widely used in food, cosmetics, and pharmaceuticals to enhance stability, control release, and reduce lipid oxidation (Kakran and Antipina 2014). The solubility of plant proteins is a crucial factor in their ability to stabilize emulsions, as higher solubility can enhance the encapsulation efficiency by promoting protein adsorption at the oil–water interface (Cano‐Medina et al. 2011; Tang et al. 2020). However, excessively high solubility can decrease the viscoelasticity of the protein films, reducing their ability to stabilize emulsions. For instance, homogenization of faba bean protein increased its solubility (from 35% to 99%) but decreased its emulsifying activity (from 27.0 to 19.7 m^2^/g) and stability due to aggregation which reduced viscoelasticity and stability (Yang et al. 2018).

The molecular structure of proteins also plays a significant role in emulsion stabilization, with certain proteins, like cruciferin, forming more rigid and stable films at the oil–water interface despite lower solubility. This is attributed to higher hydrophobicity and β‐sheet content in their secondary structure, which enhances protein–protein interactions (Yang et al. 2024). However, the structural properties of plant proteins can vary significantly based on factors such as extraction methods, pH levels, ionic strength, temperature, and plant variety (Day et al. 2022).

Even slight modifications in the physicochemical environment or the application of treatments such as heating, ultrasound, high hydrostatic pressure, or enzymatic hydrolysis can lead to changes in their interfacial properties, enhancing their effectiveness as encapsulating agents (Fernandes et al. 2024). For instance, physicochemical environment changes such as pH shifting alone or in combination with other techniques can significantly improve functional properties thereby enhancing encapsulation. In a study, pH shifting combined with PEFs was applied to soybean isolate under specific conditions (10 kV/cm, pH 11). The results showed a substantial increase in encapsulation efficiency from 54% to 77%, primarily due to plant protein modification, particularly the increase in surface hydrophobicity (Wang, Zeng, et al. 2023b).

**FIGURE 5:**
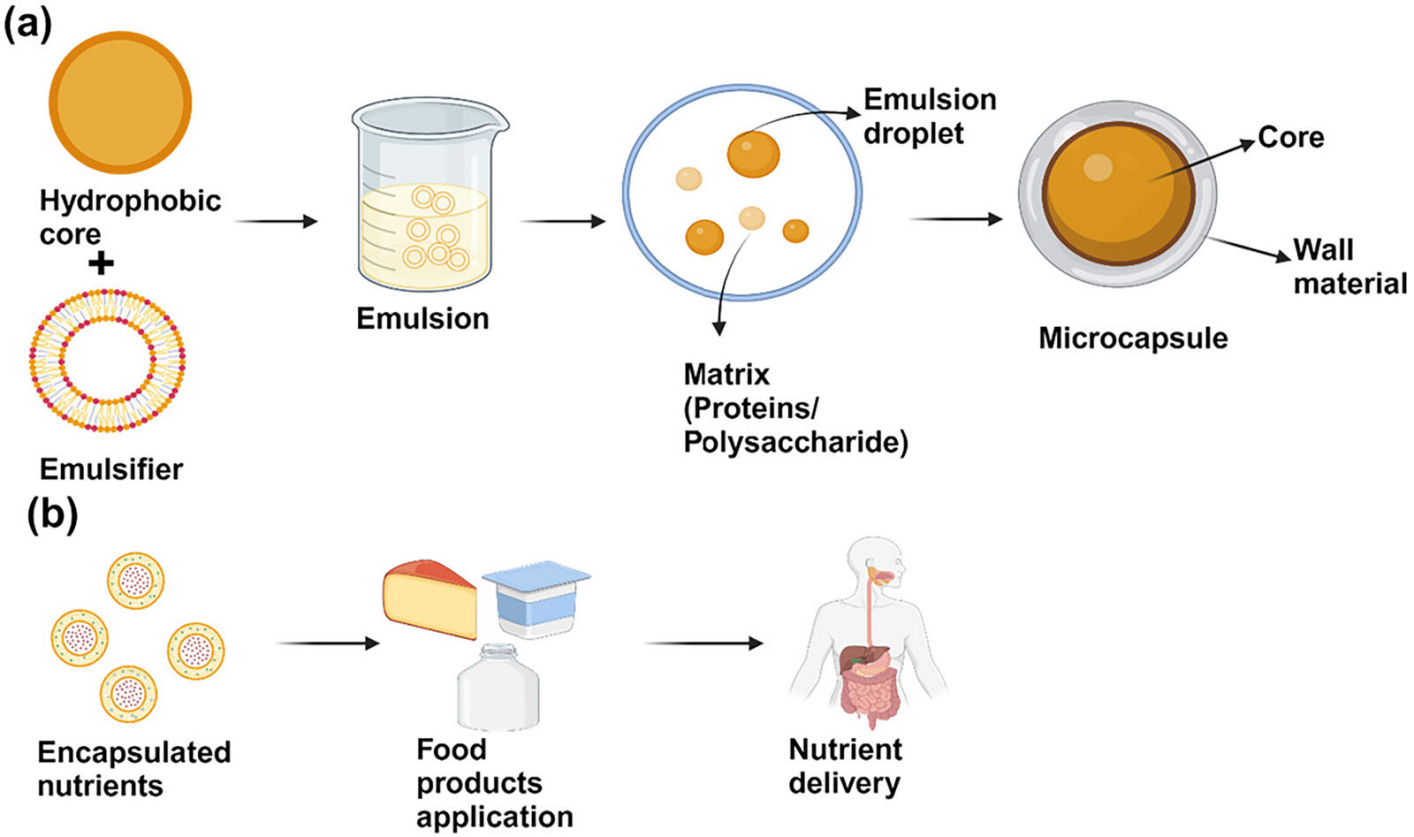
Schematic representation of (a) oil‐in‐water microencapsulation process and (b) microcapsule structure and application in food products (created in BioRender. https://BioRender.com/v43p186).

Figure 5 illustrates the process of nutrient encapsulation and delivery using plant proteins in food. These encapsulated nutrients can then be delivered to the body.

## 7 Plant Proteins for Elder Nutrition

### 7.1 Functional Comparison of Plant Proteins With Animal Proteins in the Senior Population

As the global population ages, maintaining their health becomes increasingly vital. Protein intake is crucial in preserving elders’ good health, but not all protein sources are created equal. Animal proteins are traditionally favored for their superior digestibility and amino acid profile, although plant proteins could be a suitable alternative. However, the nutritional limitations of plant proteins, including lower digestibility and deficiencies in essential amino acids (Pinckaers et al. 2021), pose challenges that need to be addressed to meet the dietary needs of the senior adults. Different modification approaches can address these issues including digestibility and bioaccessibility.

In aging populations, muscle loss is partly due to a diminished response to dietary protein. While both animal and plant proteins are used in senior diets, plant proteins often have lower digestibility and are deficient in essential amino acids like lysine and methionine. Animal proteins generally induce a stronger anabolic response but are linked to higher environmental impacts. Blending plant proteins or partially replacing animal proteins with plant‐based alternatives can help balance nutrition and sustainability (Gorissen and Witard 2018). However, evidence supporting specific intake requirements or long‐term anabolic superiority of blended approaches in senior adults remains limited and requires further well‐controlled human intervention studies.

Research shows that animal proteins, such as dairy, stimulate muscle protein synthesis more effectively than plant proteins. For instance, whey protein, which is high in leucine, is quickly digested and enhances muscle synthesis better than slowly digested proteins like casein (Burd et al. 2012). These outcomes are similar in young adults (Tang et al. 2009) and highlight the significance of high leucine concentrations in muscle protein synthesis (West et al. 2011). However, plant proteins like soy still show anabolic potential, though less than animal proteins (Wilkinson et al. 2007). These effects are largely attributed to differences in essential amino acid composition, leucine content, and digestion kinetics.

Table 4 shows a comparison of the anabolic, muscle mass, physical performance, and digestive properties of plant and animal proteins based on various studies. The table summarizes key experimental designs, methods, and outcomes, indicating that while animal proteins generally exhibit superior anabolic effects, plant proteins when consumed in adequate amounts or combinations can still provide significant benefits.

**TABLE 4:** Comparison of plant and animal protein intake in senior populations in terms of anabolism to physical performance.

Properties	Design	Method	Outcomes	References
**Anabolic**	8 males, 38–50 years, 87–102 kg, 7 females, 38–48 years, 73–83 kg consumed 24 g whey/pea protein on training days + high‐intensity functional training (4 sessions/week) 8 weeks	Bioelectrical impedance analysis	Ingestion of whey and pea protein produced similar outcomes in measurements of body composition, especially lean mass and muscle thickness	Banaszek et al. (2019)
	60 males, 70–72 years, 25 kg, 35 g wheat protein, wheat protein hydrolysate, micellar casein, whey protein, or 60 g wheat protein hydrolysate	Isotope tracer	Animal proteins have high anabolic properties as compared to plant proteins, but theoretically increasing plant proteins dose or combination of compatible profile plant proteins can be favorable for senior people	Gorissen et al. (2016)
**Digestion**	Rats (aged 20 months)	Protein Digestibility‐Corrected Amino Acid Score	Alkaline/heat‐treated soy protein isolate (49%) and lactalbumin (64%)	Gilani and Sepehr (2003)
	Standardized static in vitro digestion protocol mimicking adult and senior conditions	INFOGEST standardized static in vitro digestion protocol mimicking senior conditions	Adult gastric phase, whey (71.1%) and wheat (80.3%), while pea and rice proteins had lower digestibility at 29.9% and 15.6%. Under senior conditions, wheat protein hydrolysis remained like that of adults, but gastric proteolysis for whey, pea, and rice proteins significantly decreased to 9.5%, 14.5%, and 8.7%	Melchior et al. (2023)
	Thirty 20‐month‐old male Wistar rats	Isoproteic and isocaloric diet	Older rats utilized pea protein as efficiently as casein or whey proteins, due to its high digestibility and favorable amino acid profile	Salles et al. (2021)
**Muscle mass**	554 females, 65–72 years daily consumption cholecalciferol (800 IU; 20 µg) and Ca (1000 mg) for 3 years	Dual‐energy X‐ray absorptiometry	Higher total and animal protein intakes were associated with increased lean mass and appendicular lean mass Higher plant protein intake was associated with less reduction in appendicular lean mass	Isanejad et al. (2015)
	327 males and females, 66–76 years food‐frequency questionnaire, Bioelectrical resistance (50 kHz at 500 uA)	Bioelectrical impedance analysis	Low total and plant protein intakes were associated with a higher risk for low muscle mass	Huang et al. (2016)
	1746 men and women Food‐frequency questionnaire, Age (range: 29–85)	X‐ray absorptiometry	High intakes of total and animal protein were protective against loss of grip strength in community‐dwelling adults aged 60 years and older	McLean et al. (2016)
	997 adults [50.7% male, mean age 65.5] with a baseline gait speed ≥ 0.8 m/s Food‐frequency questionnaire	6 m walk test	The plant‐to‐animal protein intake ratio was not associated with the risk of developing slow gait speed	Huijgen et al. (2025)
	2771 older adults (≥ 65 years), appendicular skeletal muscle mass (ASM) prediction formula, cohort study	Dual‐energy X‐ray absorptiometry	Plant‐based dietary patterns reduced the risk of low muscle mass by 5%. In addition, a high plant‐based food company with a high animal‐based food intake pattern reduced the risk of low muscle mass by 60%	Ren et al. (2023)
	Participants (*n* = 171) 50 years, (plant: *n* = 60, dairy: *n* = 56, low protein: *n* = 55)	Double‐blinded, randomized, controlled	Increasing protein intake by 20 g daily for 12 weeks, whether from plant or dairy sources, improved the hand grip	Wirth et al. (2024)
	25 elders; 55–75 years, four testing sessions with standardized meal (two oz‐eq of either unprocessed lean pork, whole eggs, black beans, or sliced almonds), 300 min trials, online software (Randomization.com)	Randomized, investigator‐blind, crossover design	Pork (7.36 g EAA) and eggs (5.38 g EAA) resulted in greater essential amino acids (EAA) bioavailability than black beans (3.02 g EAA) and almonds (1.85 g EAA) in older adults. Pork resulted in greater EAA bioavailability than eggs in older adults (*p* = 0.0007)	Connolly et al. (2023)
	Thirty senior men (age 71 ± 5 years)	Short physical performance battery (SPPB)	Rates of myofibrillar protein synthesis for S20 were less than W20 (*p* = 0.02) in both exercised and nonexercised leg muscles	Yang et al. (2012)
**Physical performance**	3133 participants, 71.8 ± 4.9 years, 6‐m walking test, the mean total, plant and animal protein intakes were 1.3 ± 0.6, 0.6 ± 0.3 and 0.7 ± 0.4 g/kg BW, food‐frequency questionnaire	Linear regression	Higher plant protein intake was associated with less decline in physical performance (*β* 0.723, SE 0.288, *p* = 0.012). No associations were observed for total animal protein intakes	Yeung and Woo (2022)
	554 women aged 65.3–71·6 years	Cross‐sectional, dual‐energy X‐ray absorptiometry	Women with higher protein intake (≥ 1.2 g/kg BW) had better performance in hand‐grip strength/body mass (GS/BM; *p* = 0.001), knee extension/BM (*p* = 0.003), one‐leg stance (*p* = 0.047), chair rise (*p =* 0.043), squat (*p* = 0.019), squat to the ground (*p* = 0.001), faster walking speed for 10 m (*p* = 0.005) and higher short physical performance battery score (*p* = 0.004) compared with those with moderate and lower intakes (0.81–1.19 and ≤ 0.8 g/kg BW, respectively)	Isanejad et al. (2016)

Although plant proteins offer promising alternatives to animal proteins, while studies indicate that animal proteins generally show better anabolic and muscle mass benefits for the seniors (Connolly et al. 2023; Gorissen et al. 2016), some plant proteins, such as pea protein, have demonstrated comparable digestibility and muscle maintenance potential (Salles et al. 2021). However, plant proteins typically have lower amino acid bioaccessibility and digestibility, which may limit their effectiveness in senior adults (Melchior et al. 2023). This highlights the need for further research to enhance the functional properties of plant proteins, enabling their wider use as effective alternatives to animal proteins in senior nutrition. Strategies such as partial replacement with animal proteins and plant protein blends offer potential solutions, but further research is needed to optimize these approaches for use in products like emulsions and powders, which could deliver specific micronutrients to senior adults.

## 8 Conclusion

As the senior population grows, addressing their unique nutritional needs becomes increasingly critical. Plant proteins offer a sustainable and health‐promoting alternative to animal proteins but require extensive modifications to meet the functional demands of senior nutrition. This review highlights the importance of using physical, chemical, and enzymatic modification techniques to enhance the functional properties of plant proteins including solubility, digestibility, bioaccessibility, gelling, and nutrient‐delivery capabilities, particularly in senior dietary needs. The advancements in these modification strategies have shown that plant proteins, especially when modified appropriately, can approach or even surpass the functionality of animal proteins. This makes them viable candidates for inclusion in senior food formulations, contributing to better health outcomes by addressing issues such as muscle loss, digestion difficulties, and nutrient deficiencies. To advance the development of plant‐based foods for senior adults, future research should focus on following priorities (i) validating modified plant protein formulations against established dysphagia texture standards (e.g., IDDSI levels) to ensure safe swallowing; (ii) conducting sensory acceptance and palatability testing specifically in senior adult populations to assess flavor, texture, and overall acceptability; (iii) evaluating the scalability, cost‐effectiveness, and processing feasibility for industrial translation; and (iv) assessing the impact of modifications on protein digestibility and amino acid bioaccessibility, particularly in the context of age‐related anabolic resistance. By doing so, we can better support the health and well‐being of the aging population through more effective and tailored nutritional interventions.

## Author Contributions

**Kinza Mukhtar**: conceptualization, methodology, investigation, formal analysis, visualization, writing – original draft. **Jian Ying**: writing – review and editing, investigation, methodology. **Yong Wang**: conceptualization, methodology, formal analysis, supervision, funding acquisition, visualization, project administration, writing – review and editing. **Cordelia Selomulya**: conceptualization, writing – review and editing, methodology, funding acquisition, project administration, resources, supervision.

## Conflicts of Interest

The authors declare no conflicts of interest.

## Data Availability

No data were generated.
